# Diesel exhaust induces gut microbiome dysbiosis and reduced fecal acetate: Role of acetate supplementation

**DOI:** 10.1016/j.ecoenv.2025.118654

**Published:** 2025-08-04

**Authors:** Rajat Gupta, Candace Chang, Laurent Vergnes, Dawoud Sulaiman, Fen Yin, James A. Stewart, Margarete Mehrabian, Joel D. Kaufman, Jonathan P. Jacobs, Aldons J. Lusis, Karen Reue, Michael E. Rosenfeld, Jesus A. Araujo

**Affiliations:** a Division of Cardiology, David Geffen School of Medicine, University of California Los Angeles, Los Angeles, CA, USA; b Environmental and Molecular Toxicology Interdepartmental Program, University of California Los Angeles, Los Angeles, CA, USA; c Department of Environmental Health Sciences, Fielding School of Public Health, University of California Los Angeles, Los Angeles, CA, USA; d Vatche and Tamar Manoukian Division of Digestive Diseases, David Geffen School of Medicine, University of California Los Angeles, Los Angeles, CA, USA; e Department of Human Genetics, David Geffen School of Medicine, University of California Los Angeles, Los Angeles, CA, USA; f Department of Environmental and Occupational Health Sciences, University of Washington, Seattle, WA, USA; g Division of Gastroenterology, Hepatology and Parenteral Nutrition, Veterans Administration Greater Los Angeles Healthcare System, Los Angeles, CA, USA; h Goodman-Luskin Microbiome Center, University of California Los Angeles, Los Angeles, CA, USA; i Department of Microbiology, Immunology and Molecular Genetics, David Geffen School of Medicine, University of California Los Angeles, Los Angeles, CA, USA; j Molecular Biology Institute, University of California Los Angeles, Los Angeles, CA, USA; k Department of Pathology, University of Washington, Seattle, WA, USA

**Keywords:** Dyslipidemias, Lipoxygenase, Triglycerides, Mitochondrial dysfunction, Particulate matter, Short chain fatty acids, Gut microbiome

## Abstract

Air pollution exposure enhances the risk of cardiovascular morbidity and mortality. Epidemiological studies provide strong evidence of a link between exposure to ambient particulate matter with aerodynamic diameter< 2.5 μm (PM_2.5_) and development of cardiovascular and metabolic disorders. We have shown that inhaled ultrafine particles (UFP) or whole diesel exhaust (DE), enriched in UFP, induce cardiometabolic effects, including dyslipidemia and hepatic steatosis. However, the pathogenic mechanisms remain unknown. We recently demonstrated that exposure to ambient particulate in the ultrafine-size range altered the gut microbiota composition in various animal models, with a potential to induce systemic effects. Thus, we hypothesized that sub-chronic inhalation exposure to DE leads to gut dysbiosis and altered gut-derived metabolites, likely responsible for some of the metabolic effects. Male apolipoprotein E^−/−^ (ApoE^−/−^) mice, exposed to inhaled DE *vs.* filtered air (FA) (6 h/day, 5 days/week for 16 weeks) displayed alterations in cecal microbiota composition, which associated with elevated plasma cholesterol and triglycerides, as well as hepatic triglycerides and oxidized lipids. DE exposure upregulated hepatic mRNA and protein levels of 12-lipoxygenase (Alox12), together with significantly reduced fecal acetate levels, correlating with changes in lipids and cecal microbiota composition. Metabolic effects were recapitulated in HepG2 cells treated with DE particles, including elevated *Alox12* mRNA levels and decreased respiration in isolated mitochondria. Supplementation with gut-derived short chain fatty acid acetate reversed these effects in cells. In conclusion, inhaled DE induced gut microbiome dysbiosis, lipid peroxidation and triglyceride accumulation, likely *via* mitochondrial dysfunction, which was rescued in cells by acetate supplementation.

## 1. Introduction

Exposure to ambient air pollution was responsible for 9 million deaths worldwide in 2019, a majority of which were due to cardiovascular disease (Murray, 2020; Rajagopalan and Landrigan, 2021). Epidemiological studies have shown that exposure to the particulate matter (PM) component of air pollution, with an aerodynamic diameter equals to or smaller than 2.5 μm (PM_2.5_) strongly correlates with air pollution-induced cardiometabolic effects (Bell et al., 2017; J. Li et al., 2019; Mathew et al., 2018; L. Wang et al., 2021). Mechanistic studies in mouse models of cardiovascular disease have also revealed that chronic exposure to ultrafine particles (UFP) resulted in systemic cardiovascular (CV) and metabolic effects including hyperlipidemia and worsened atherosclerosis (Araujo et al., 2008; R. Li et al., 2013).

A common source of air pollution in urban settings is particles derived from diesel-powered motor vehicles. Indeed, diesel exhaust (DE) comprises of hundreds of constituents in either gas or particulate form, including PM_2.5_ enriched in UFP (Zheng et al., 2007). We have previously shown that whole body exposure to freshly generated, aged, diluted DE for only 2 weeks in apolipoprotein E^−/−^ (ApoE^−/−^) mice on a chow diet resulted in increased lipid peroxidation in the liver as judged by elevated levels of 5-hydroxyeicosatetraenoic acid (5-HETE), *via* activation of the 5-Lipoxygenase pathway, with loss of plasma high-density lipoprotein (HDL) anti-inflammatory properties (Yin et al., 2013). In addition, ApoE^−/−^ mice exposed to inhaled DE for 16 weeks also exhibited increased lipid peroxidation, as evidenced by increased hepatic levels of 9- and 13-hydroxyoctadecadienoic acid (HODEs) instead, which are stable oxidation products of linoleic acid, along with elevated plasma and hepatic triglycerides, likely due to mitochondrial dysfunction, as judged by our *in-vitro* work with HepG2 cells (Yin et al., 2019) and by demonstration *in-vivo* after only 2 weeks of exposure to DE (Ramanathan et al., 2024). However, the underlying pathogenic mechanisms of how DE or PM in general promote these adverse prooxidative and systemic metabolic effects remain poorly understood.

Ambient air pollution exposure has been shown to alter the human gut microbiota composition, with a potential to promote cardiometabolic and other adverse health effects (Filardo et al., 2022; Jonsson and Backhed, 2017). Indeed, there is mounting evidence to suggest a strong link between changes in the gut microbiome and the development of a wide variety of human diseases, including cardiovascular diseases (Tang and Hazen, 2014), metabolic disorders (Hur and Lee, 2015), cancer (Algrafi et al., 2023), as well as neurological disorders (Hur and Lee, 2015; Tang and Hazen, 2014; Tiwari et al., 2023). It is postulated that inhaled PM or its chemical constituents could access the gastrointestinal tract *via* mucociliary clearance to the oropharynx followed by swallowing (Kreyling et al., 1999), or through ingestion of PM deposited on food and water (Beamish et al., 2011). We and others have shown that exposure to ambient PM by inhalation or oral gavage markedly altered the gut microbiota composition (Chang et al., 2024; Kish et al., 2013; Mutlu et al., 2018; W. Wang et al., 2018), that associated with atherogenic lipid metabolites in the plasma, suggesting the gut-vascular transmissibility of prooxidative and metabolic effects (Li et al., 2017). Furthermore, oral administration of diesel exhaust particles (DEP) induced gut dysbiosis together with a dose-dependent reduction in the levels of short chain fatty acids (SCFAs) (van den Brule et al., 2021), which are gut-derived metabolites from anaerobic fermentation of dietary fibers, and mainly consist of acetate, propionate, and butyrate. Importantly, previous studies have shown that SCFAs play a protective role against high fat diet-induced obesity (Shimizu et al., 2019), as well as regulating lipid metabolism (He et al., 2020; Shah et al., 2021), in association with reduced risk of CV and metabolic diseases (Bartolomaeus et al., 2019; Chambers et al., 2018). Nonetheless, it is still not clear whether DE exposure by inhalation could promote gut microbiota dysbiosis and/or alterations in SCFAs, that might contribute to cardiometabolic effects.

In this study, we examined whether chronic inhalation of whole DE in chow fed ApoE^−/−^ mice promotes alterations in the gut microbiota composition and microbial metabolites, that might be associated with systemic prooxidative and metabolic effects. Furthermore, we also explored mechanistic plausibility and evaluated the potential of SCFA acetate to inhibit DEP-induced effects on lipid peroxidation as well as mitochondrial dysfunction *via* an *in-vitro* approach using HepG2 cells.

## 2. Materials and methods

The authors declare that all supporting data are available within the article and Appendix A. Supplementary material.

### 2.1. Diesel exhaust exposure system

Details on the diesel exhaust exposure system characteristics have been described previously (Fox et al., 2015; Gould et al., 2008; Yin et al., 2019, 2013). For additional details, please refer to Appendix A. Supplementary material.

### 2.2. Animals and exposure protocol

Mice were exposed to DE *vs.* Filtered Air (FA) by whole body inhalation for 16-weeks as described previously in detail (Yin et al., 2019). Male ApoE^−/−^ (Strain #:002052, B6.129P2-*Apoe*^*tm1Unc*^/J) mice on a C57BL/6 J background were shipped from The Jackson Laboratory (Bar Harbor, Me) and kept in a controlled environment with a 12-h light/dark cycle. During non-exposure periods, mice were housed in regular mouse cages and allowed access to autoclaved water and standard chow (#5053, Lab Diets, St. Louis, MO) *ad libitum*. At 31 weeks of age, mice were moved into enclosed Allentown racks and randomly assigned to two groups (n = 8–9/group) that were exposed for 16 weeks to either: 1) DE (experimental group), or 2) Filtered air (control group), with minimal stress during exposures. Mice were housed in a total of 4 cages (2 cages/group) with 3–5 mice/cage. Following a 7-day acclimatization period, exposures were started in the Allentown racks with each session comprising of 6 h/day and 5 days/week. Following the last exposure session, blood was drawn after overnight fasting, from the retro-orbital sinus of each mouse, and other tissues including the liver and whole intestines were harvested for conducting downstream analysis. All animal procedures were approved by the University of Washington Institutional Animal Care and Use Committee.

### 2.3. Plasma and hepatic lipids

Plasma levels of cholesterol and triglycerides, as well as liver triglycerides and oxidized free fatty acids were measured in our previous study (Yin et al., 2019). For additional details, please refer to Appendix A. Supplementary material.

### 2.4. Sample preparation and 16S rRNA gene sequencing

Following euthanasia, contents of the cecum and small intestine were carefully scraped out into a sterile tube using a flat spatula and the bacterial DNA was isolated by utilizing the PowerSoil DNA Isolation Kit (MO BIO Labs). The sequencing of 16S ribosomal RNA gene, and phylogenetic profiling were done as described previously (Org et al., 2015). In brief, the bacterial primers 515 F and 806 R were used to amplify and sequence the V4 hypervariable region of the 16S ribosomal RNA gene, with amplicons generated for each of the cecal and small intestinal samples per mouse and were optimized for Illumina MiSeq. Only biological replicates were used, and each unique sample was only sequenced once. For additional details, please refer to Appendix A. Supplementary material.

### 2.5. Analysis of microbiome diversity

Alpha (α) diversity was analyzed using several indices including Shannon index (measuring species evenness and richness), Chao1 index (measuring species richness), and Average Faith’s index (distance between taxa on the phylogenic tree) with data rarefied to the lowest number of sequence depth in R. For additional details, please refer to Appendix A. Supplementary material.

### 2.6. Preparation of diesel exhaust particles organic extract

Diesel exhaust particles (DEP) was provided as a generous gift, originally produced at the National Institute of Environmental Studies (Tsukuba, Ibaraki, Japan). Its chemical composition has been previously characterized, including polycyclic aromatic hydrocarbons (PAHs) and quinone content (Gong et al., 2007; N. Li et al., 2004; N. Li et al., 2002). A methanol extract of the DEP (DEPe) was prepared as recently described (Ramanathan et al., 2024). Briefly, DEP was resuspended in methanol, sonicated on ice, and centrifuged. The supernatant was dried under nitrogen gas, the extract was dissolved in DMSO in the dark and stored at −80°C until use.

### 2.7. Tissue culture

The human HepG2 hepatocellular carcinoma cell line was obtained from American Type Culture Collection (ATCC, Manassas, VA, USA), and cultured in DMEM media containing 10 % fetal bovine serum (Hyclone, Logan, UT), along with 1 % penicillin-streptomycin (Invitrogen, Carlsbad, CA). For additional details, please refer to Appendix A. Supplementary material.

### 2.8. Gene expression analysis

Total mRNA was prepared, and quantification of gene expression levels were done using real-time polymerase chain reaction (RT-PCR) as previously described (Araujo et al., 2008; Yin et al., 2019). For additional details, please refer to Appendix A. Supplementary material.

### 2.9. Immunoblots

Mouse livers were harvested, and the tissue lysates were generated by centrifugation at 14,000 ×g for 10 min at 4°C. Concentrations of proteins were estimated using the bicinchoninic acid assay kit (Pierce, Rockford, IL). 30 μg protein was electrophoresed on a 10 % denaturing polyacrylamide stacking gel and then transferred to a PVDF membrane (BIO-RAD, Hercules, CA) at 4°C. After blocking with 5 % non-fat milk, membranes underwent incubation with a rabbit polyclonal anti-ALOX12 antibody (PA5–26020, Thermo Fisher Scientific, CA) at 4°C overnight. They were rinsed in PBS/0.1 % Tween 20 and further incubated with goat anti-rabbit HRP conjugated secondary antibody (sc-2004, Santa Cruz Biotechnology, CA). The band intensities were normalized to GAPDH. Densitometry was performed using ImageJ analysis.

### 2.10. Quantification of short chain fatty acids

Mouse fecal samples were extracted and analyzed for SCFAs by direct-injection gas chromatography (GC) at the Center for Human Nutrition in UCLA as described previously (Zhao et al., 2006). For additional details, please refer to Appendix A. Supplementary material.

### 2.11. Mitochondrial bioenergetics

Assessment of mitochondrial oxygen consumption rate (OCR) was performed by utilizing an XF24 or XF96 Analyzer (Agilent) as reported previously (Yin et al., 2019). For additional details, please refer to Appendix A. Supplementary material.

### 2.12. Statistical analyses

Data were reported as means ± SEM. If the data passed the Shapiro Wilk’s normality test, then analysis was conducted by parametric tests, including unpaired Student’s *t* test for comparison of data between 2 groups, or 1-way ANOVA followed by Tukey’s post hoc test for comparison of data in ≥ 3 groups. On the other hand, if the normality test failed, then data was analyzed with nonparametric tests, including Mann Whitney *U* test for comparison of data between 2 groups, or Kruskal-Wallis test followed by Dunn’s post hoc test for comparison of data in ≥ 3 groups. Associations between changes in the gut microbiota composition and lipids were analyzed by non-parametric linear regression analysis using Spearman’s correlation coefficients. All other associations were analyzed using parametric linear regression analysis using Pearson’s correlation coefficients, calculated with GraphPad Prism 9.5.1 software for Windows. The type of statistical test used for each experiment or figure were specified in the corresponding figure legends. *p* ≤ 0.05 were considered statistically significant. For microbiome analysis, adjustment of *p*-values for multiple hypothesis testing were done using the Benjamin–Hochberg method, with a significance threshold set at *p.adj* < 0.05. All microbiota data were adjusted for cage effects unless specified otherwise.

## 3. Results

### 3.1. Exposure to inhaled DE altered microbial abundance in the gut

**Fig. 1.**
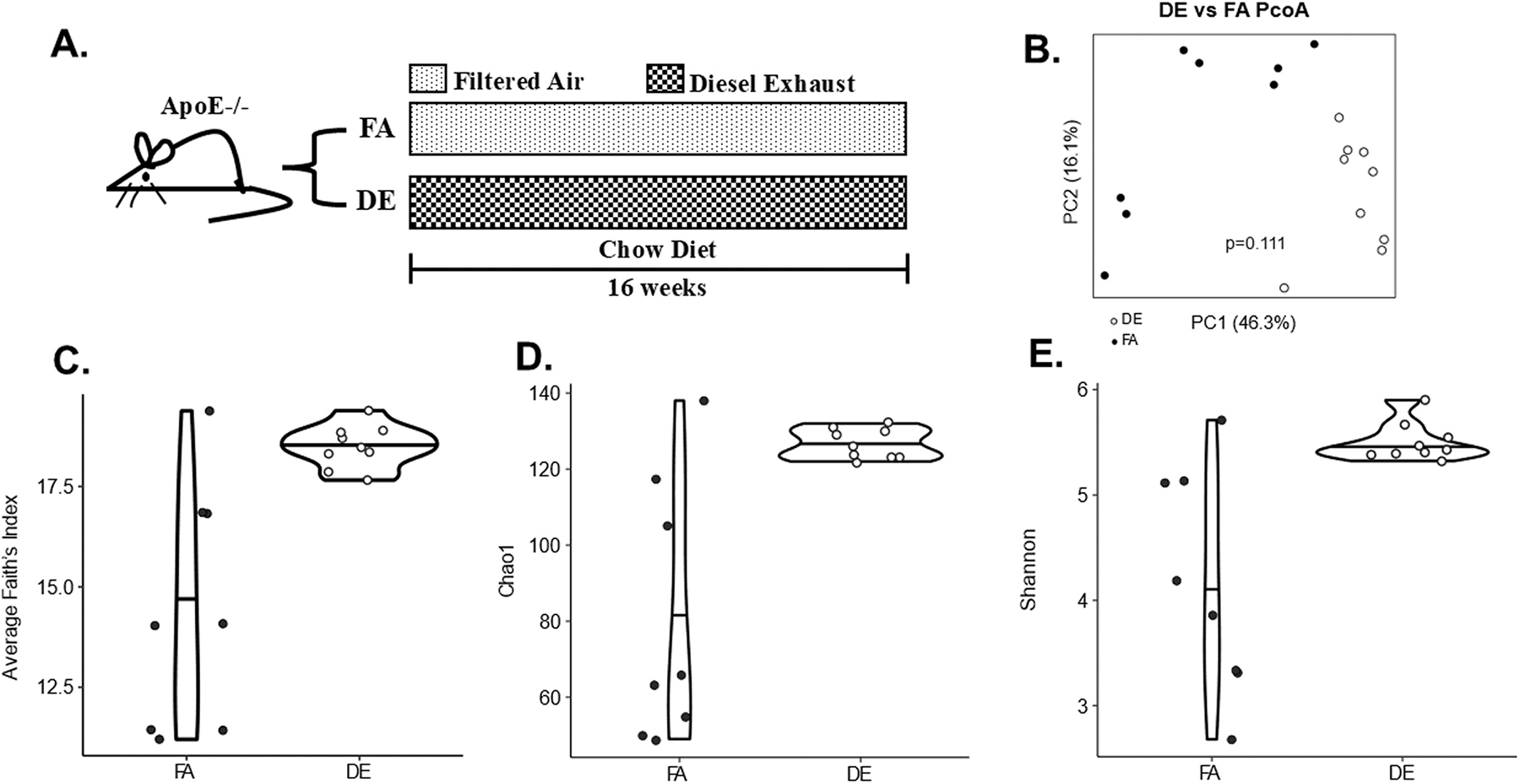
DE exposure and gut microbial diversity. (A) Schematic diagram, (B) β-diversity, by principal coordinates analysis (PCoA) of Bray-Curtis dissimilarity. Significance was assessed by permutational multivariate analysis of variance (PERMANOVA). (C-E) α-diversity using Average Faith’s index (p = 0.31) (C), Chao1 (p = 0.34) (D), and Shannon indices (p = 0.34) (E). Significance was determined by a linear mixed effects model with adjustment for cage effects. Each dot represents one sample with color representing the exposure group (DE or FA). n = 8–9/group.

To investigate the effects induced by inhaled DE on gut microbial profiles, we evaluated microbial diversity within and between groups through α- and β-diversity indices, respectively, as well as the relative abundance of bacteria in the cecum and small intestine of male ApoE^−/−^ mice exposed to inhaled DE *vs.* FA for 16 weeks (Fig. 1A). Bray Curtis dissimilarity was used to assess β-diversity, while α-diversity was determined by Chao1, Shannon and Average Faith’s indices. β-diversity analysis showed differences in cecal microbiota composition with DE inhalation by PERMANOVA (p = 0.02), and univariate α-diversity analyses showed increased cecal microbial diversity in the DE inhalation group by the Average Faith’s index (p = 0.006), Chao1 index (p = 0.006) and Shannon index (p = 0.009) (Fig. S1). However, these differences lost statistical significance after adjustment for cage effects (Fig. 1B-E) as described in the methods. In addition, no significant differences were observed in β-diversity (Fig. S2) or α-diversity in the small intestine, between the DE and FA exposed groups (Fig. S3).

**Fig. 2.**
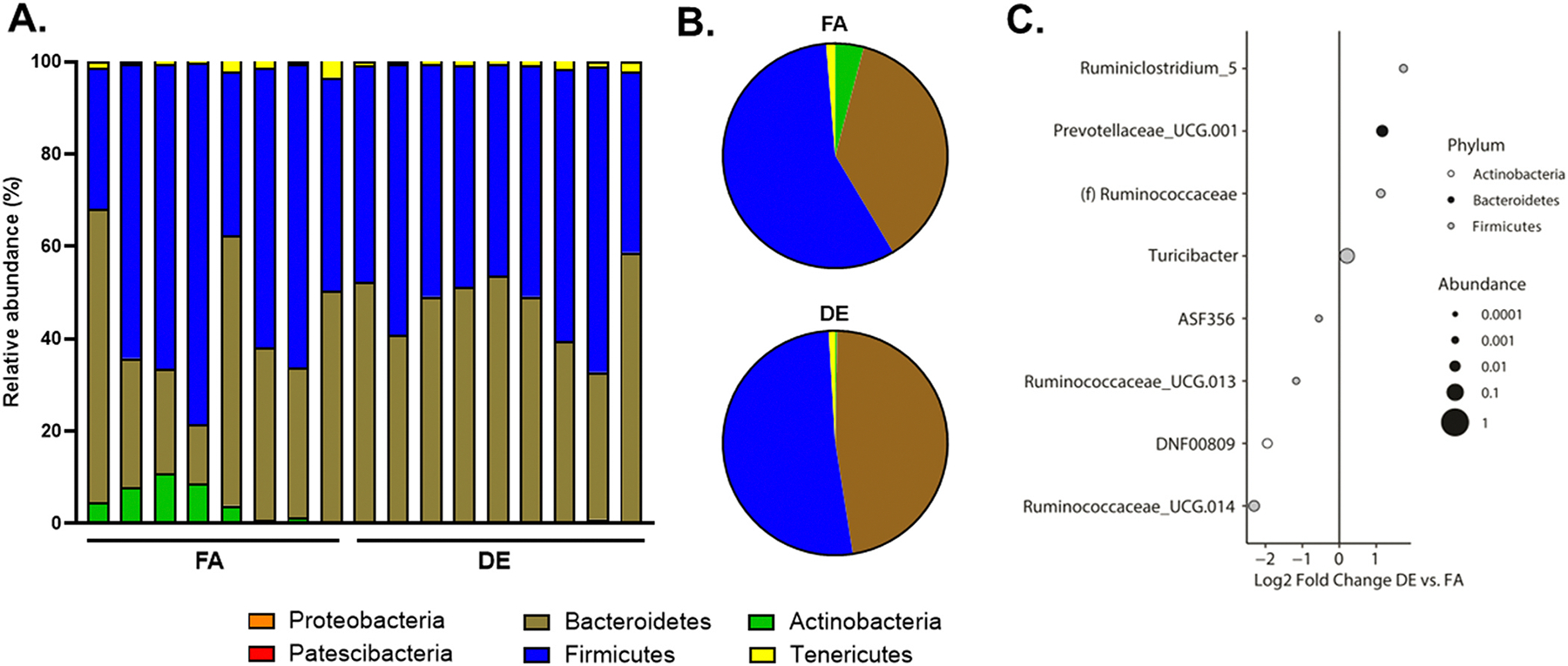
DE exposure and gut microbiota composition and abundance. 16S rRNA sequencing of cecal DNA was performed to characterize bacterial composition. (A-B) Differences in %relative abundance of cecal bacteria at the phylum level. Phyla with > 1 % mean abundance were included. (C) Log2 fold changes of cecal bacteria with statistically significant differences between DE and FA exposed groups. n = 8–9/group. Dot size is proportional to the relative abundance of bacteria and color represents the phylum. The magnitude of effect is shown as the log2 of the fold change (Log2 Fold Change) between the DE and FA exposed groups. n = 8–9/group.

On the other hand, microbiota composition analysis demonstrated alterations in multiple bacterial phylum in mice exposed to DE *vs.* FA (Fig. 2A-B). Thus, after cage effects and multiple comparisons adjustment, DE exposure resulted in a statistically significant expansion of *Ruminiclostridium_5, Prevotellacaeae_UCG.001, Ruminococcaceae,* and *Turicibacter*, while *Ruminococcaceae_UCG.014, DNF00809, Ruminococcaceae_UCG.013,* and *ASF356* were significantly reduced, as shown by log2 fold change levels in Fig. 2C. DE exposure also led to microbial compositional differences in the small intestine including significant increase in *Akkermansia*, *Ruminococcaceae_UCG.009* and *Erysipelatoclostridium* (Fig. S4), indicating differential effects of DE *vs.* FA exposure on microbial taxonomic abundance in both the cecum and small intestine.

### 3.2. DE exposure increased circulating cholesterol and triglyceride levels in association with changes in cecal microbiome composition

**Fig. 3.**
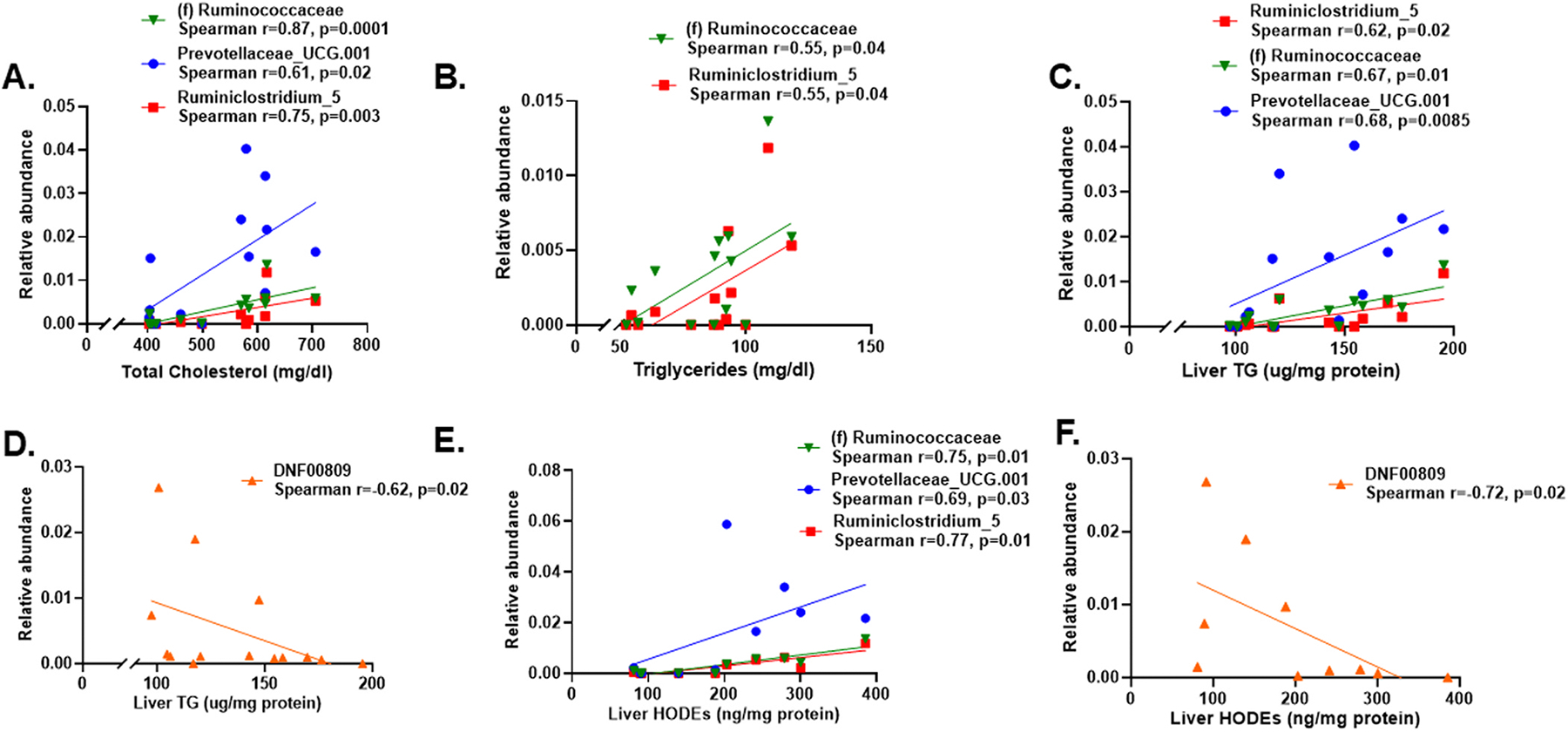
Associations between plasma and hepatic lipids, and cecal microbiome. Spearman’s correlations between cecal microbiota abundance and (A) plasma total cholesterol, and (B) plasma triglycerides. (C) Spearman’s correlations indicating a positive association or (D) negative association between hepatic triglycerides and cecal microbiome abundance at the genus level. (E) Spearman’s correlations indicating a positive association or (F) negative association between hepatic total HODEs (9-HODE + 13-HODE) and cecal microbiome abundance at the genus level, among the DE and FA exposed groups. Correlation coefficient (r) and *p* values for each significant microbial taxa are indicated within the figure. HODEs, hydroxyoctadecadienoic acids.

We have previously reported that DE exposure for 16 weeks led to increased plasma levels of total cholesterol and triglycerides in ApoE^−/−^ mice fed a chow diet (Fig. S5) (Yin et al., 2019). We further conducted Spearman’s correlation analysis to determine if there were associations between circulating lipids and microbes that were differentially abundant between DE and FA exposures. Interestingly, there were significant positive associations between plasma total cholesterol levels and genus-level cecal microbiota-*Prevotellaceae_UCG.001* (r = 0.61, p = 0.02), *Ruminiclostridium_5* (r = 0.75, p = 0.003) and *(f) Ruminococcaceae* (r = 0.87, p = 0.0001) (Fig. 3A). Likewise, there were significant positive associations between plasma triglyceride levels and *Ruminiclostridium_5* (r = 0.55, p = 0.04) and *(f) Ruminococcaceae* (r = 0.55, p = 0.04) (Fig. 3B) but no negative associations between cecal microbiota abundance and circulating lipids.

### 3.3. Changes in cecal microbiome composition associated with alterations in oxidized fatty acids and triglycerides in the liver

**Fig. 4.**
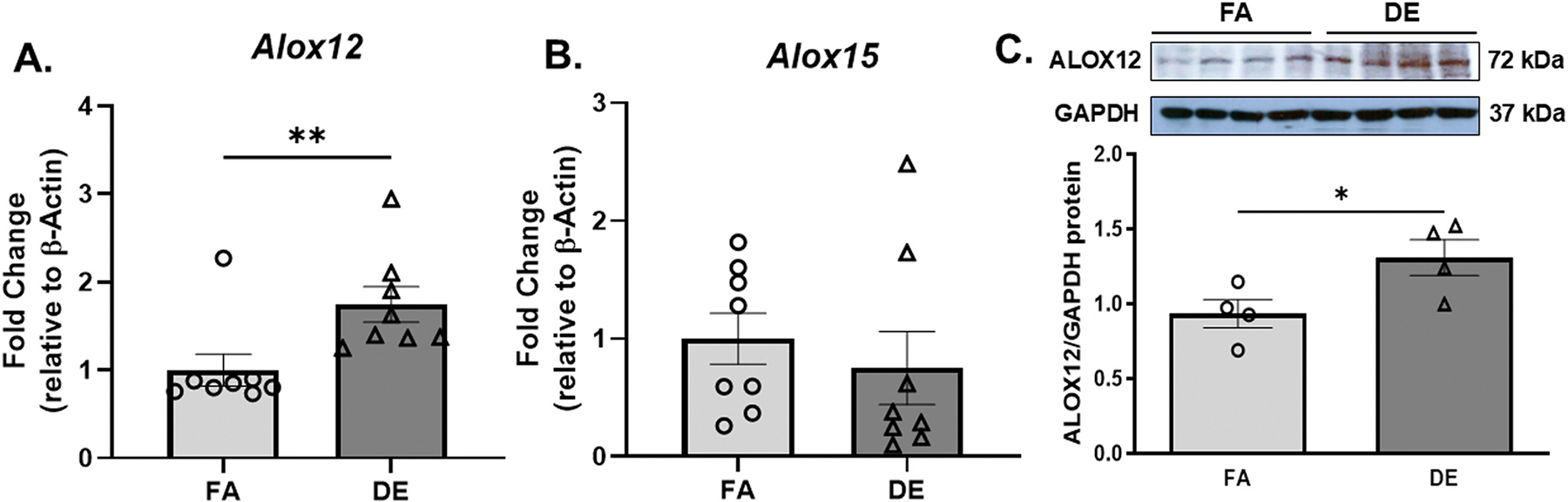
DE exposure and hepatic 12-lipoxygenase mRNA and protein levels. Effect of inhaled DE *vs.* FA exposure on hepatic mRNA levels of (A) *Alox12* and (B) *Alox15* as determined by fold change relative to β-actin. (C) ALOX12 protein levels in the liver after DE *vs*. FA exposure as measured by immunoblotting and quantified using densitometric analysis by ImageJ. **p < 0.01, DE *vs.* FA using Mann-Whitney *U* test (Panel (A)) and *p < 0.05, DE *vs.* FA using unpaired Student’s *t*-test (Panel (C)). n = 8/group for panels (A) and (B), and n = 4/group for panel (C).

Exposure to DE for 16 weeks also increased hepatic triglyceride levels as compared with FA exposure (Fig. S6A) (Yin et al., 2019). We further observed significant associations between liver triglycerides and cecal microbiota abundance that were either positive [*Prevotellaceae_UCG.001* (r = 0.68, p = 0.0085), *Ruminiclostridium_5* (r = 0.62, p = 0.02) and *(f) Ruminococcaceae* (r = 0.67, p = 0.01)] (Fig. 3C), or negative [*DNF00809* (r = −0.62, p = 0.02)] (Fig. 3D). Moreover, exposure to DE significantly elevated hepatic levels of total hydroxyoctadecadienoic acids (HODEs: 9 +13-HODE) (Fig. S6B), which derive from the oxidation of linoleic acid (LA). Interestingly, significant correlations were observed between liver total HODEs and cecal microbiota abundance that were either positive [*Prevotellaceae_UCG.001* (r = 0.69, p = 0.03), *Ruminiclostridium_5* (r = 0.77, p = 0.01) and *(f) Ruminococcaceae* (r = 0.75, p = 0.01)] (Fig. 3E), or negative [DNF00809 (r = −0.72, p = 0.02)] (Fig. 3F). DE-induced hepatic 9-HODE and 13-HODEs that were significantly associated with changes in cecal microbiota (Fig. S7). Thus, our data indicates that DE-induced alterations in cecal microbiota occurred together with elevations in hepatic triglycerides and HODEs. We then asked whether an increase in the levels of 9- or 13-HODEs could have been associated with activation of lipoxygenases, known to catalyze oxidation of LA to HODEs. Thus, we measured mRNA expression of 12- and 15-lipoxygenase (*Alox12, Alox15*). While DE exposure did not increase hepatic *Alox15* mRNA levels, there was a significant upregulation in *Alox12* mRNA and protein levels (Fig. 4), suggesting the possibility that activation of *Alox12* could have mediated, at least in part, DE prooxidative effects in the liver.

### 3.4. DE exposure inhibited fecal acetate levels in association with changes in cecal microbiome composition and lipids

**Fig. 5.**
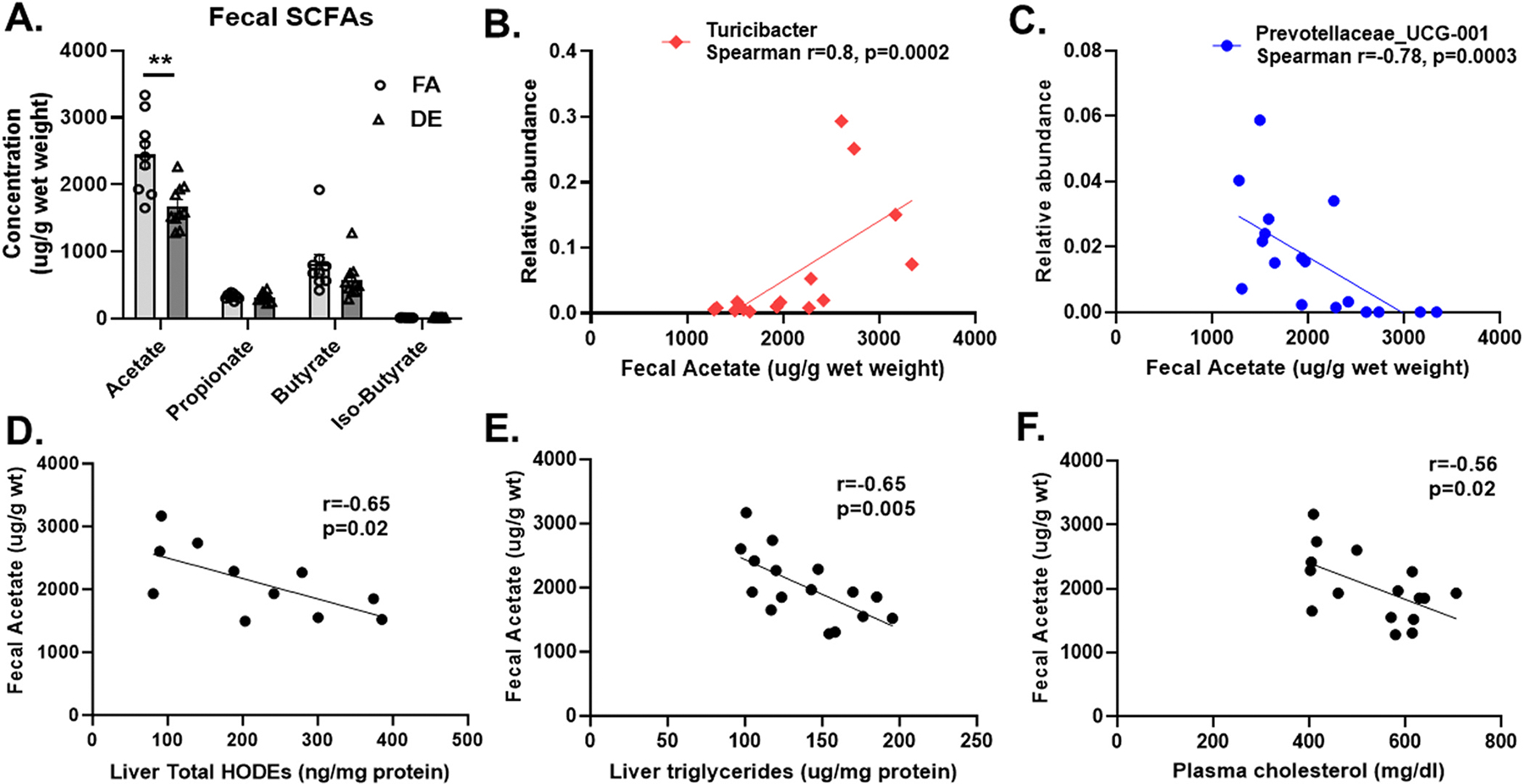
Fecal short chain fatty acids, cecal microbiome and lipids. (A) Fecal levels of short chain fatty acids (SCFAs), (B) Spearman’s rank correlation indicating a positive association or (C) negative association between fecal acetate and cecal microbial taxa abundance at the genus level. Pearson’s correlation indicating a negative association between fecal acetate and (D) hepatic HODEs, (E) hepatic triglycerides and (F) plasma cholesterol, among the DE and FA exposed groups. Each bar denotes mean ± SEM (n = 8–9/group), **p < 0.01 DE vs. FA controls using Student’s *t*-test (Panel (A)).

We asked how DE exposure was able to induce effects in the liver and turned our attention to gut microbial metabolites such as SCFAs. Interestingly, DE exposure led to significantly reduced fecal levels of acetate as compared to FA (p < 0.01), but without affecting the fecal concentrations of other SCFAs (Fig. 5A). There were no significant differences in the levels of SCFAs in the plasma (data not shown). We also observed significant correlations between fecal acetate levels and cecal microbiota abundance that were either positive [*Turicibacter* (r = 0.8, p = 0.0002) (Fig. 5B), *Ruminococcaceae_UCG-013* (r = 0.67, p = 0.0039) and *DNF00809* (r = 0.822, p = 0.0001) (Fig. S8A)], or negative [*Prevotellaceae_UCG.001* (r = −0.78, p = 0.0003) (Fig. 5C), *Ruminiclostridium_5* (r = −0.49, p = 0.04) and *(f) Ruminococcaceae* (r = −0.61, p = 0.0099) (Fig. S8B)]. In addition, fecal acetate levels were negatively correlated with hepatic levels of 9- and 13-HODEs (Fig. S9), total HODEs (Fig. 5D) and triglycerides (Fig. 5E), as well as plasma levels of cholesterol (Fig. 5F), suggesting the possibility that the reduction in acetate could have contributed to the DE-induced increases in HODEs and triglycerides in the liver.

### 3.5. Acetate supplementation inhibited DEP effects in HepG2 cells

**Fig. 6.**
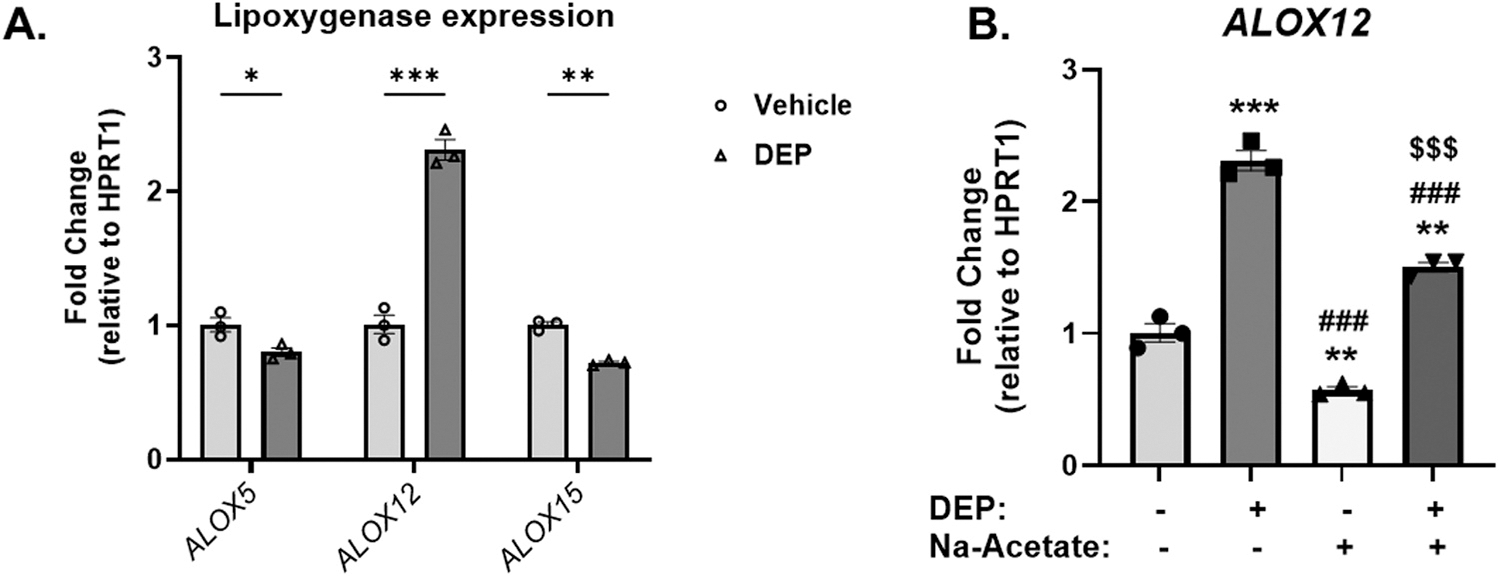
Lipoxygenase expression and effect of acetate supplementation on *ALOX12* mRNA levels induced by diesel exhaust particles in HepG2 cells. (A) mRNA levels of *ALOX5*, *ALOX12* and *ALOX15*, after treatment with vehicle control or whole diesel exhaust particles (DEP) of HepG2 cells for 4 h. (B) mRNA levels of *ALOX12*, after treatment with vehicle, diesel exhaust particles (DEP) and DEP + Sodium Acetate (Na-Acetate) for 4 h following Na-Acetate pre-treatment for 16 h in HepG2 cells. ***p < 0.001, **p < 0.01, *p < 0.05 *vs.* vehicle control; ^###^p < 0.001, ^##^p < 0.01 *vs.* DEP; ^$ $ $^p < 0.001, ^$ $^p < 0.01 *vs.* Na-acetate, analyzed using 1-way ANOVA and Tukey post hoc test. Statistical significance in panel (A) was determined using multiple Student’s t test with Welch’s correction.

To test mechanistic plausibility of inhaled DE with the possibility of chemical constituents entering the systemic circulation, we used an *in-vitro* approach that previously helped us to identify DEP effects on mitochondrial function and decreased β-fatty acid oxidation using HepG2 cells (Yin et al., 2019). In this study, we evaluated whether treatment with DEP would recapitulate the increases in *Alox12* observed *in vivo* and tested if supplementation with acetate would inhibit it. Thus, HepG2 cells were treated with or without sodium acetate (10 mmol/L for 16 h) and then incubated them with DEP (100 μg/ml for 4 h). Consistent with our *in-vivo* observations, DEP treatment significantly upregulated *ALOX12* mRNA levels, but downregulated *ALOX5* and *ALOX15* mRNA levels instead, indicating that DEP-induced prooxidative effects could be due to activation of the 12-lipoxygenase pathway (Fig. 6A). Interestingly, pre-treatment with sodium acetate significantly inhibited DEP induction of *ALOX12* mRNA levels (Fig. 6B).

**Fig. 7.**
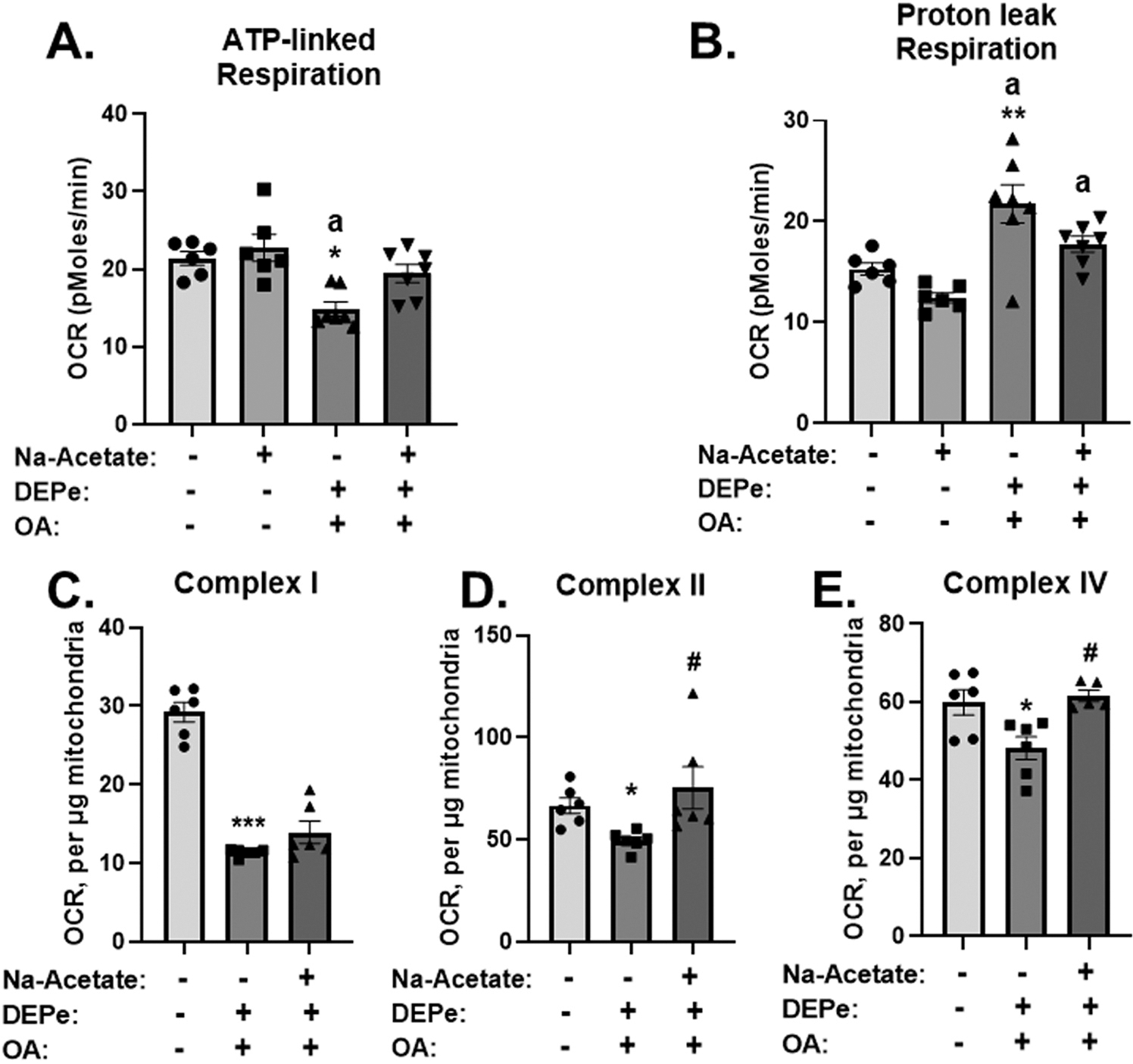
Acetate supplementation rescued cellular respiration and inhibited mitochondrial dysfunction. HepG2 cells were treated with vehicle or 100 μg/ml of DEP extract (DEPe) for 4 h followed by 22 h of 2 mmol/L oleic acid (OA) treatment. Cells were pre-incubated with 2 mmol/L sodium acetate (Na-Acetate) for 1 h before the DEPe+OA treatment and for the remaining time. Oxygen consumption rate (OCR) was measured using an XF analyzer to assess cellular mitochondrial respiration. (A) ATP-linked, and (B) Proton leak respiration. Mitochondrial complexes I, II and IV oxygen consumption rate (OCR) was measured in mitochondria isolated from mouse liver and treated for 1 h with vehicle or 150 μg/ml of DEP extract (DEPe), and 3 mmol/L oleic acid (OA). Mitochondria were pre-incubated with 1 mmol/L sodium acetate (Na-Acetate) for 30 min before the DEPe+OA treatment and for the remaining time. (C) Complex I, (D) Complex II, (E) Complex IV activity. *p < 0.05, ***p < 0.001 *vs.* vehicle control; ^a^p < 0.05 *vs.* acetate only, ^#^p < 0.05 *vs.* DEPe+OA analyzed using Kruskal-Wallis test and Dunn’s post hoc test, except for panel (B) where **p < 0.01 *vs.* vehicle control; ^a^p < 0.05 *vs.* acetate only, and panel (E) where *p < 0.05 *vs.* vehicle control; ^#^p < 0.05 *vs.* DEPe+OA were analyzed using 1-way ANOVA and Tukey’s post hoc test.

We also tested whether acetate supplementation could suppress other DEP metabolic effects by administering it to cells prior to a co-treatment with an organic extract of DEP (DEPe) + oleic Acid (OA), which we have shown that it decreases cellular respiration indicative of mitochondrial dysfunction, with impairment in β-oxidation and accumulation of fatty acids, as well as increased cellular content of triglycerides (Yin et al., 2019). Thus, our data showed that DEPe + OA significantly decreased ATP-linked respiration, suggesting a defect in ATP production (Fig. 7A), and markedly increasing proton leak respiration (Fig. 7B) consistent with our previous report (Yin et al., 2019). Importantly, both of these effects were rescued by acetate even though acetate alone did not alter any of these parameters (Fig. 7A and B). Next, we investigated whether acetate supplementation could alleviate DEPe-induced impairment of mitochondrial function directly on isolated mitochondria. Fresh mitochondria were isolated from mouse liver and treated with DEPe + OA for 1 h, with or without a pretreatment of acetate for 30 mins. DEPe + OA co-treatment markedly suppressed mitochondrial respiration consistent with our previous report (Yin et al., 2019). Remarkably, acetate co-treatment with DEPe + OA significantly rescued complex II and complex IV respiration, which remained similar to the vehicle control but did not rescue the decrease in complex I respiration induced by DEPe + OA (Fig. 7C-E). Altogether, these results demonstrated that acetate supplementation ameliorated the mitochondrial damage induced by DEPe + OA.

## 4. Discussion

Here we demonstrate that ApoE^−/−^ mice subjected to long-term inhalation of DE exhibited changes in the gut microbiota composition, in association with metabolic effects including hepatic steatosis and hyperlipidemia. In addition, DE exposure upregulated *Alox12* mRNA and protein levels, and induced lipid peroxidation (9- and 13-HODEs) in the liver, together with a reduction in fecal acetate levels. *In-vitro* work with HepG2 cells enabled us to demonstrate that acetate supplementation rescued DEP effects on *ALOX12* upregulation and mitochondrial dysfunction, suggesting the possibility that deficiency in acetate levels could have contributed, at least in part, to the metabolic effects induced by DE.

To investigate pathogenic mechanisms mediating DE-induced lipid peroxidation and hyperlipidemia, we asked whether chronic exposure to whole DE altered the gut microbiota composition in association with adverse health outcomes. Our study is the first to demonstrate changes in the gut microbiota composition after inhalation of DE, a physiologically relevant route of PM exposure, which could promote gut-vascular transmissibility of metabolic effects. Indeed, our *in-vivo* data showed that inhaled DE markedly altered the gut microbiota composition (Fig. 2 and S4), in association with changes in the levels of plasma and hepatic lipids (Fig. 3 and S7). Unexpectedly, no statistically significant differences were present in α- and β-diversity of the microbiota in the cecum (Fig. 1B-E) and small intestine (Fig. S2 and S3) due to cage effects, which are known to artificially confound variations in the gut microbiota (Laukens et al., 2016) due to mixing with coprophagia (Campbell et al., 2012; Kim et al., 2017) when mice are not housed individually. However, we did observe alterations in the cecal microbiota composition, that remained statistically significant after adjustment for cage effects, together with various metabolic alterations indicating DE-induced development of gut dysbiosis. These findings importantly extend a previous study showing that oral ingestion of DEP, rich in ultrafine particles, altered the gut microbiota composition (van den Brule et al., 2021), in a similar manner how both oral ingestion (R. Li et al., 2017) and inhalation of ambient ultrafine particles (Chang et al., 2024) induced gut dysbiosis as well. Furthermore, inhalation of PM_2.5_ (W. Wang et al., 2018) and oral ingestion of PM_10_ (Kish et al., 2013) have also been shown to induce gut dysbiosis indicating that particulate matter of all sizes and various sources have the ability of influencing the gut microbiome.

How does DE or its chemical constituents influence the gut microbiome after inhalation exposure? DE gaseous or particulate components could have accessed the GI tract by swallowing of the gases or particles deposited in the lower epithelial airways that are swept back by mucociliary clearance or through oral ingestion *via* contaminated food or water (Beamish et al., 2011). Alternatively, the gut microbiome could have been impacted by changes in the oral intake and dietary pattern, as it has been reported that inhaled DE particulate can regulate expression of genes related to appetite in the hypothalamus, resulting in increased weight gain and obesity (H. Liu et al., 2024), and subsequent changes in the gut microbiota composition (H. Liu et al., 2024). While we cannot rule out this possibility since we did not record food consumption or evaluated expression of hypothalamic appetite-related genes, it is unlikely since DE-exposed mice did not exhibit a significant weight gain, but only a trend as compared to FA-exposed mice (p = 0.18, Fig. S10).

We observed a marked reduction in fecal acetate levels, which are the most abundant SCFAs produced in the gut, that significantly correlated with changes in cecal microbiome and other lipids (Fig. 5 and Fig. S8). SCFAs are gut-derived metabolites produced from the bacterial fermentation of dietary fiber in the GI tract and mainly comprises of acetate, propionate and butyrate, which are considered important in regulating lipid metabolism and oxidative stress signaling pathways (He et al., 2020; P. Liu et al., 2021). In our study, we observed that fecal levels of propionate and butyrate remained unaltered after inhalation of DE, contrary to previous studies where a reduction in other SCFAs were observed with either oral ingestion of PM_10_ or DEP (Kish et al., 2013; van den Brule et al., 2021), which could be due to differences in the route of PM exposure and/or type of rodent model used. On the other hand, inhalation of motor vehicle exhaust for 24 weeks in a rat model of chronic obstructive pulmonary disease (COPD) induced gut dysbiosis, and led to significantly reduced colonic levels of acetate (N. Li et al., 2020), consistent with our findings. It is well known that several microbial species are producers of acetate including *Ruminococci*, *Prevotella* and *Akkermansia muciniphila* among many others (Akhtar et al., 2022; Richards et al., 2016). Indeed, we noticed a significant reduction in *Ruminococcaceae_UCG.013* and *Ruminococcaceae_UCG.014* in the cecum (Fig. 2C), but at the same time of a significant enrichment in other acetate producing taxa such as *Ruminococcaceae (f)* and *Prevotellaceae_UCG.001* in the cecum, and *Akkermansia* and *Ruminococcaceae_UCG.009* in the small intestine of DE-exposed mice (Fig. 2C and Fig. S4A). Therefore, it is possible that the decrease in some acetate-producing taxa may have outweighed the enrichment of other acetate producers, resulting in a net overall decrease in its levels (Fig. 5A). On the contrary, acetate consumption for further metabolism, particularly for the production of butyrate by certain gut bacteria such as *Faecalibacterium duncaniae*, could also lead to lower levels of acetate (Verstraeten et al., 2024). Furthermore, certain *Bacteroides* species, such as *Bacteroides thetaiotaomicron*, can also consume acetate and other sugars, like glucose, to fuel their fermentation processes, potentially impacting host carbohydrate availability (Wong et al., 2024). However, we did not observe a significant enrichment in commonly known acetate consuming bacteria and therefore, less likely that decreased acetate levels could be attributed to enrichment in these species. This supports the need for greater taxonomic resolution coupled with functional analysis by future studies to identify the specific taxa of cecal microbiota altered by DE and which ones, could be producers or consumers of acetate.

We then asked whether changes in microbial taxa resulting in reduced production of acetate could have contributed to some of the metabolic effects induced by DE. Our data shows that fecal acetate levels negatively correlated with liver HODEs (Fig. 5D and S9), triglycerides (Fig. 5E), and plasma cholesterol (Fig. 5F). Acetate has been shown to promote beneficial metabolic effects as demonstrated by reduced levels of hepatic and plasma triglycerides in rabbits after subcutaneous administration of acetate for 4 days, due to decreased synthesis and increased catabolism of fatty acids (L. Liu et al., 2019). Acetate has also been shown to improve hepatic mitochondrial function and reduce *de novo* lipogenesis in the liver, with a therapeutic potential to mitigate fatty liver disease (Sahuri-Arisoylu et al., 2016). These results are consistent with our data suggesting that deficiency in acetate could have contributed, at least in part, to DE effects on liver triglycerides and HODEs, as well as plasma cholesterol and triglycerides (Fig. S5 and S6). In addition, the gut microbiome can affect lipid production through several potential pathways, including alterations in hepatic lipid metabolism, cholesterol transport and bile acid metabolism, *via* its various metabolites such as SCFAs, trimethylamine N-oxide (TMAO) and bile acids (Jia et al., 2021). For example, dietary SCFA intake has been reported to exert metabolic benefits to the host by suppressing high fat diet-induced liver weight gain and hepatic triglyceride accumulation (Shimizu et al., 2019). In addition, the gut microbial metabolite TMAO is closely linked to host cholesterol and bile acid metabolism, as it reduces the expression of *Cyp7a1*, which is involved in synthesis of bile acids, with subsequent inhibition of cholesterol transport and increased accumulation of cholesterol by the cells (Pathak et al., 2020). It is also known that *Ruminiclostridium* can influence bile acid metabolism primarily through the deconjugation of bile salts, which can ultimately affect the composition of bile acid pool and other metabolic processes (Thompson et al., 2021). In addition, gut microbiota can also change the integrity of intestinal epithelial cells and the intestine, regulate lipid storage in adipose tissue and promote lipid oxidation in muscles, thereby contributing to changes in lipid metabolism (Backhed et al., 2004; Rohr et al., 2020).

DE exposure also led to activation of the 12-lipoxygenase (12-LOX) pathway in the liver as evidenced by a significant increase in hepatic mRNA and protein levels of *Alox12* in DE-exposed mice (Fig. 4). This is important since increased *Alox12* could have contributed to elevated levels of 9- and 13-HODEs in the liver, which are stable oxidation products of linoleic acid (LA). Indeed, murine 12/15-lipoxygenase (12/15-LOX), a mouse orthologue of human 15-lipoxygenase-1 (15-LOX-1), converts LA into 13(S)-HODE and 9(S)-HODE (Singh and Rao, 2019), that were increased in liver biopsy samples obtained from nonalcoholic steatohepatitis (NASH) subjects (Hall et al., 2017). Importantly, it has been shown that 12/15-LOX plays a detrimental role in promoting hepatic steatosis, insulin resistance, and inflammation in hyperlipidemic mice since genetic disruption of 12/15-LOX protected these animals from nonalcoholic fatty liver disease (NAFLD) (Martinez-Clemente et al., 2010). Our *in-vitro* data in HepG2 cells indicates a significant upregulation in the mRNA levels of *ALOX12* but not *ALOX5* or *ALOX15*, after treatment with whole DEP (Fig. 6A) and likely triggered lipid peroxidation *via* activation of the *Alox12* pathway, similar to our findings *in-vivo* where DE exposure led to increased mRNA and protein levels of *Alox12* (Fig. 4). While it is likely that increased Alox12 could have contributed to elevated HODEs, we did not characterize their geometric isomers and therefore, cannot rule out that those HODEs elevations could have derived from non-enzymatic reactions. Interestingly, two-week exposure to whole DE led to increased mRNA expression of *Alox5*, but not *Alox12* or *Alox15*, together with increased levels of 5-hydroxyeicosatetraenoic acids (5-HETEs) in the liver, indicative of 5-Lipoxygenase pathway activation (Yin et al., 2013), and evidence of mitochondrial dysfunction (Ramanathan et al., 2024), suggesting that various lipoxygenase pathways can be activated by DE with different time kinetics. In addition, there is increasing evidence that air pollution exposure in general leads to activation of various lipoxygenase pathways in humans as well (Lin et al., 2022, 2019).

To determine the potential of DE particles to activate *ALOX12* by direct interaction with hepatocytes, we utilized HepG2 cells which exhibited *ALOX12* activation after incubation with DEP for 4 h (Fig. 6A), consistent with findings *in-vivo*. These cells have also been instrumental to model *in-vitro*, DE steatotic effects occurring *in-vivo*, when DEP is administered together with oleic acid (OA) (Yin et al., 2019). We administered acetate to HepG2 cells prior to their incubation with DEP +/− OA and evaluated *ALOX12* expression and mitochondrial function to explore the potential of acetate to counteract DEP-induced effects. Indeed, acetate supplementation markedly reduced *ALOX12* mRNA upregulation in comparison to DEP treatment alone (Fig. 6B), and rescued ATP-linked and proton leak (p = 0.09) respiration in HepG2 cells, as well as activity of complexes II and IV in isolated mitochondria from mouse livers (Fig. 7). These findings offer indirect support to the notion that reduced acetate could have also contributed to some of the effects induced by DE *in-vivo*. Interestingly, other studies have reported direct effects of SCFAs on *ALOX5* and *ALOX12* activation in the presence of butyrate *via* histone acetylation in human intestinal epithelial cells (Ohata et al., 2005; Wachtershauser et al., 2000). In addition, acetic acid consumption for 8–12 weeks in the form of vinegar was reported to lower circulating levels of triglycerides in subjects with obesity or hyperlipidemia (Beheshti et al., 2012; Kondo et al., 2009), indicating the therapeutic potential of acetate to improve lipid metabolism and mitochondrial dysfunction (Sahuri-Arisoylu et al., 2016).

## 5. Limitations

Our study has several limitations. Firstly, our assessment of the gut microbes did not resolve the individual microbial species affected within the genera and it appears that several of these effects could be exhibited by various members of the same genera. Secondly, previous studies have demonstrated a causal role of altered gut microbiota due to dietary exposure to environmental toxicants such as microplastics (polystyrene microspheres) in mediating inflammation and other systemic effects, as demonstrated by fecal microbiota transplantation experiments after antibiotics treatment (Zhai et al., 2024). In our study, although we did not test for causality, our data suggests the likelihood of altered gut microbial taxa, in contributing at least in part, to DE-induced metabolic effects, which will be addressed by future studies. Thirdly, while we performed adjustment for cage effects and statistically significant taxa, the associations between microbiome abundance and metabolic parameters were not adjusted for multiple comparisons. Importantly, we adjusted for cage effects to account for the interindividual variability in mice related to factors, including early life environment, baseline microbiome and husbandry etc. This variability arises from a complex interplay of environmental factors such as dietary intake as well as grouped housing. Unfortunately, we did not measure differences in daily food intake in our mice, which could have led to variations in the gut microbiome as well as fecal SCFAs. In addition, the inclusion of additional controls such as individual housing in metabolic cages to define temporal food intake patterns could have been beneficial for this study, even though single housing introduces its own concerns due to social stress on the mice, which could further impact the gut microbiome. Lastly, our study only assessed male mice in the ApoE^−/−^ background; therefore, future studies should include females as well as mice on a C57BL/6 background to determine the potential for sex and genetic strain differences in microbial effects.

## 6. Conclusions and future directions

Long-term exposure to inhaled DE led to alterations in cecal microbiome composition that associated with oxidative effects, hyperlipidemia, and hepatic steatosis. As a result, DE exposure led to reduced availability of acetate, which rescued DEP-induced *ALOX12* upregulation and mitochondrial dysfunction, *via* its supplementation to HepG2 cells. Overall, our data suggests that reduced availability of acetate due to gut dysbiosis may contribute to gut-vascular transmissibility of DE-induced oxidative and metabolic effects. In the future, we plan to conduct cecal microbiota transplantation experiments using either antibiotic-treated ApoE^−/−^ mice and/or germ-free gnotobiotic ApoE^−/−^ mice to examine whether the DE-induced metabolic effects can be mediated *via* the gut microbiome. In addition, we will also examine whether acetate supplementation in ApoE^−/−^ mice might inhibit the cardiometabolic toxicity induced by exposure to DE. Lastly, we will also characterize sex specific effects on changes in the gut microbiota composition and/or its metabolites induced by exposure to air pollution.

## Supplementary Material

1

## Data Availability

Data will be made available on request.

## Appendix A. Supporting information

Supplementary data associated with this article can be found in the online version at doi:10.1016/j.ecoenv.2025.118654.

## Abbreviations:

Alox5: 5-Lipoxygenase
Alox12: 12-Lipoxygenase
Alox15: 15-Lipoxygenase
ApoE^−/−^: Apolipoprotein E Knockout
ASV: Amplicon Sequence Variants
DE: Diesel Exhaust
DEP: Diesel Exhaust Particles
DEPe: Diesel Exhaust Particles Organic Extract
FA: Filtered Air
LA: Linoleic Acid
OA: Oleic Acid
OCR: Oxygen Consumption Rate
PERMANOVA: Permutational Multivariate Analysis of Variance
PM: Particulate Matter
SCFAs: Short Chain Fatty Acids
UFP: Ultrafine Particles
