# Supplementary material for: Diesel exhaust induces gut microbiome dysbiosis and reduced fecal acetate: Role of acetate supplementation
Source: Ecotoxicol Environ Saf. Author manuscript; Available in PMC 2026 May 31. (PMC13222647; doi:10.1016/j.ecoenv.2025.118654)
Supplement: 1 [file NIHMS2170834-supplement-1.docx]

**Diesel Exhaust Induces Gut Microbiome Dysbiosis and Reduced Fecal Acetate:**

**Role of Acetate Supplementation**

Rajat Gupta ^a, b, c^, Candace Chang ^a, b, c, d^, Laurent Vergnes ^e^, Dawoud Sulaiman ^a^, Fen Yin ^a^­­, James A. Stewart ^f^, Margarete Mehrabian ^a^, Joel D. Kaufman ^f^, Jonathan P. Jacobs ^b, d, g, h^,

Aldons J. Lusis ^a, e, i, j^, Karen Reue ^e^, Michael E. Rosenfeld ^f, k^, Jesus A. Araujo ^a, b, c, j, *^

^a^Division of Cardiology, David Geffen School of Medicine, University of California Los Angeles, Los Angeles, California, USA.

^b^Environmental and Molecular Toxicology Interdepartmental Program, University of California Los Angeles, Los Angeles, California, USA.

^c^Department of Environmental Health Sciences, Fielding School of Public Health, University of California Los Angeles, Los Angeles, California, USA.

^d^Vatche and Tamar Manoukian Division of Digestive Diseases, David Geffen School of Medicine, University of California Los Angeles, Los Angeles, California, USA.

^e^Department of Human Genetics, David Geffen School of Medicine, University of California Los Angeles, Los Angeles, California, USA.

^f^Department of Environmental and Occupational Health Sciences, University of Washington, Seattle, Washington, USA.^g^Division of Gastroenterology, Hepatology and Parenteral Nutrition, Veterans Administration Greater Los Angeles Healthcare System, Los Angeles, California, USA.

^h^Goodman-Luskin Microbiome Center, University of California Los Angeles, Los Angeles, California, USA.

^i^Department of Microbiology, Immunology and Molecular Genetics, David Geffen School of Medicine, University of California Los Angeles, Los Angeles, California, USA.

^j^Molecular Biology Institute, University of California Los Angeles, Los Angeles, California, USA.

^k^Department of Pathology, University of Washington, Seattle, Washington, USA.

**Running Title: Air Pollution and Dysbiosis**

***Corresponding author**: Jesus A. Araujo, MD, PhD. Division of Cardiology, Department of Medicine, David Geffen School of Medicine, University of California-Los Angeles, 10833 Le Conte Avenue, CHS 43-264, Los Angeles, CA 90095. P.O. Box 951679. Phone number (310) 825-3222, Fax number (310) 206-9133. E-mail address: JAraujo@mednet.ucla.edu

**Materials and methods**

**Diesel exhaust exposure system**

Briefly, diesel exhaust (DE) was derived from a 5.5 kW, single-cylinder generator Yanmar model YDG5500EV-6EI). The engine load selected by setting a load bank (Simplex, Model Swift-E FT, Springfield, IL) to 4.0 kW ≈72 % generator output. We used ultralow sulfur diesel fuel from local fuel distributors with a maximum sulfur content of 15 ppm. The lubricating oil for the generator was Chevron DELO 400LE, SAE 15 W-40. A separate cage rack, which delivered HEPA filtered air to the cages using air drawn from the animal housing room was used for the filtered air (FA) control exposures. The DE cage rack drew diluted emissions from a point in the exposure system just upstream of the main exposure room. The air entering the exposure room was conditioned to 18°C and 60% relative humidity. Approximately 6-8 mins of aging occured between DE dilution and the entrainment into the animal housing racks. The resulting environment in DE exposures hence represented freshly generated, diluted, and aged model of traffic-related air pollution. We continuously measured the concentrations of particulates during exposures and maintained them at steady levels using a feedback controller monitoring fine particulate levels.

The mass concentration of DE was monitored during exposures with a TEOM analyzer (Rupprecht & Patashnick Model 1400a) and an integrating light-scattering nephelometer (Radiance Research Model 903) (Gould et al., 2008). DE was titrated to achieve a concentration of 220 μg/m^3^ PM_2.5_ mass (198-242 μg/m^3^). Operation of the engine under the same conditions used here has resulted in quite reliable DE emissions, mimicking the characteristics reported in detail in our previous study (Yin et al., 2013) including: a) mass fraction of particle-bound polycyclic aromatic hydrocarbons (PAHs) of 21 ng/µg PM_2.5_ (SD 2); (b) ratio of the organic to elemental carbon in the particles of 0.10 (SD 0.02); (c) concentrations of oxides of nitrogen of 1220 ppb NO (SD 160) and 45 ppb NO_2_ (SD 17).

**Plasma lipids**

Plasma levels of cholesterol and triglycerides were measured in our previous study (Yin et al., 2019). Briefly, total cholesterol and triglycerides in the plasma were measured *via* colorimetric assays as per the manufacturer’s instructions (Thermo Scientific, Middletown, VA and Cayman Chemical Company, Ann Arbor, MI, respectively).

**Hepatic triglycerides**

Hepatic triglyceride content was measured in our previous study (Yin et al., 2019). Briefly, liver homogenates were prepared using 0.5M Tris (pH 7.4) and 1% Triton X-100 buffer, and triglyceride levels were then determined using the colorimetric assay used for plasma (Cayman Chemical Company, Ann Arbor, MI). Concentration of hepatic triglycerides was normalized by total protein content in the homogenates.

**Hepatic oxidized free fatty acids**

Hepatic oxidized free fatty acids were measured in our previous study (Yin et al., 2019). Briefly, mouse liver tissues (50-70 mg) were snap frozen and homogenized with 8 volumes of ice-cold Tris-HCl (40 mM, pH 7.4 with 0.02% BHT) buffer, spiked with internal standards (15(S)-HETE-d8,13(S)-HODE-d4, 50 ng/mL each, 12.5 μL in methanol). Samples were centrifuged at 6,000 g, for 15 min at 4°C, and an aliquot (about 100μL, normalized by protein concentration) was adjusted to pH ~ 3.0 using 450 μL of 0.5% acetic acid. After an incubation time of 15 min on ice, samples were then loaded onto a preconditioned solid-phase extraction (SPE) cartridge (1 cc, Waters Oasis HLB), previously equilibrated with 1 mL of methanol, followed by 1 mL of water prior to loading the sample. The cartridge was washed with 1 mL of 5% methanol and then finally eluted with 1 mL of 100% methanol. The eluate was completely evaporated using a stream of nitrogen. Samples were re-suspended using 61 μL of ethanol followed by centrifugation at 13,200 rpm for 20 min at 4°C. 20 μL aliquot of the supernatant was then analyzed by LC–MS/MS, with multiple reaction monitoring using the following m/z transitions: 295.0→194.8 for 13-HODE; 295.0→171.0 for 9-HODE. Concentrations of each analyte in the sample were estimated using a series of standards that were simultaneously prepared in water with a range of amounts of unlabeled standards and the same amount of internal standards.

**16S rRNA gene sequencing**

The conditions for PCR were: 94^o^C for 3 min; followed by 35 cycles of 94^o^C for 45 s, 50^o^C for 30 s, and 72^o^C for 90 s, and 72^o^C for 5 min. Bacterial ribosomal RNA genes underwent high-throughput sequencing analysis on the Illumina MiSeq platform (Illumina). The DADA2 pipeline (Callahan et al., 2016) in R studio was used for de-multiplexing the 16S rRNA gene sequences, yielding an average length of 250 bases per read. Sequences were binned into operational taxonomic units (OTU) and quality control was performed against the SILVA 132 reference database. Sequence depth ranged from 14,573 to 65,051 reads/sample with a mean of 24,660 for the 32 samples, divided across 8 cecal and 8 small intestinal samples from FA-exposed mice, and 9 cecal and 7 small intestinal samples from DE-exposed mice.

**Analysis of Microbiome diversity**

The α-diversity data was fitted on a linear mixed effects model (lmer) using the REML (Restricted Maximum Likelihood), with t-test using Satterthwaite’s method (Satterthwaite, 1946), where α-diversity indices are dependent variables, treatment (FA or DE) are independent variables, and mouse cages are the random effect in the model. Beta (β) diversity analysis was carried out using permutational multivariate analysis of variance (PERMANOVA) integrated in the adonis package in R (Anderson, 2014), with treatment (DE or FA) as fixed effects and cage as strata. The Bray-Curtis dissimilarity matrix was used to study differences in microbiome composition between DE and FA. Microbiota composition at the phylum level were analyzed by importing .biom files and taxa with <1% abundance across treatment groups were omitted. Differential abundance analysis between treatment groups were analyzed using glmmTMB package (Brooks et al., 2017) in R studio. Zero-inflation and non-zero inflation models were constructed before choosing the most suitable model based on comparison of Akaike information criterion (AIC) with mouse cage as a random effect. Amplicon sequence variants (ASVs) were filtered if not present in at least 25% of all samples in β-diversity and differential abundance analyses. Significantly abundant taxa were visualized using *ggplot* in R Studio. Significance threshold was set at q-value<0.05.

**Tissue Culture**

HepG2 cells were grown in sub-confluent monolayers in an incubator at 37°C, in a humidified atmosphere of 5% CO_2_ and 95% air. For gene expression assays, HepG2 cells were treated with 100 µg/ml whole DEP for 4 h with or without 10 mM sodium acetate, along with a pretreatment for 16 h. After treatment, HepG2 cells were rinsed 2-3 times with sterile PBS and harvested for mRNA isolation and gene expression analysis.

**Gene Expression analysis**

Briefly, RNA was extracted from mouse livers and HepG2 cells using TRIZOL (Invitrogen, Carlsbad, CA). cDNA was prepared by utilizing the cDNA Synthesis kit (Applied Biosystems). RT-PCR was conducted using a LightCycler 480 instrument (Roche Molecular Biochemicals), by following the manufacturer’s instructions. The PCR conditions were as follows: 95ºC for 10 min, followed up 45 cycles of 95ºC for 10 s, 60ºC for 30 s and 72ºC for 15 s. The quantity of cDNA for each gene was estimated by utilizing the 2^-ΔΔCp^ method constructed from the cycle thresholds or crossing point (C_p_) values of each dilution sample (Livak & Schmittgen, 2001). RT-PCR was conducted using the Universal Probe Library (Roche Molecular Biochemicals) and Taqman reagents (Thermofisher Scientific). Relevant information regarding the primers, probes and Taqman assays IDs are outlined in **Table S1**.

**Quantification of short chain fatty acids**

Briefly, samples were thawed, carefully weighed, and homogenized in acidic distilled H_2_O (pH 2.0) at 100 mg/mL (w/v).  The suspension was centrifuged at 10,000 g for 15 min at 4°C, after which the supernatant was pipetted to a clean tube and then spiked with an internal standard 2-ethylbutyric acid.  SCFAs were measured by gas chromatography flame ionization detection (Agilent 7890A) and StabilWAX-DA column (Restek corp. 30m×0.25mm i.d. 0.25 µm). The flow rate of Helium that was supplied as the carrier gas was 1 mL/min. The initial temperature of the oven was 95°C, that was maintained for 30 s, and further raised to 200°C at 8°C/min, then again increased to 260°C at 10°C/min, that was finally held at 260°C for 5 min.  The temperatures of the flame ionization detector (FID) and the injection port were 240 and 260°C, respectively. The flow rates of gases including air, nitrogen and hydrogen were 300, 25 and 30 mL/min, respectively. The volume of sample injected for analysis of GC was 1 μL, and each analysis run time was 24.625 min. Data handling was carried out with an HP ChemStation. Individual calibration curves were obtained for each SCFA by plotting the ratio of peak areas of individual SCFA to internal standard against the concentration of the individual SCFA and fit by linear regression. The concentration of SCFAs were calculated based on the calculation curves by using peak area ratio of sample peak against the internal standard.

**Mitochondrial Bioenergetics**

HepG2 cells underwent treatment with 100 µg/ml of an organic extract of diesel exhaust particles (DEPe) for 4 h followed by 22 h of 2 mmol/L oleic acid (OA) treatment to examine cellular OCR as described previously (Yin et al., 2019). For the measures in presence of acetate, cells were pre-incubated with 2 mmol/L acetate for 1 h, followed by co-incubation with DEPe and OA for the remaining time. Measurement for OCR was performed directly from freshly isolated mitochondria from a mouse liver as previously described (Rogers et al., 2011; Yin et al., 2019). For the assessment of β-oxidation, mitochondria treatment was performed with 80 µmol/L palmitoyl-carnitine/1 mmol/L malate (fatty acid oxidation-driven complex I) in Mitochondrial Assay Solution buffer (in the absence of glucose) and vehicle, 3 mmol/L OA+150 µg/mL DEPe for 1 h at 37ºC. For the measures in presence of acetate, cells were pre-incubated with 1 mmol/L acetate for 30 min, followed by co-incubation with DEPe and OA for the remaining time. OCR was determined prior to and after sequential injections as previously described (Yin et al., 2019).

**Table S1**: qPCR Primer sequences, UPL probes and Taqman gene expression assay IDs. UPL, University Primer Library (Roche).

| **Gene** | **Forward Seq (5’-3’)** | **Reverse Seq (5’-3’)** | **UPL Probe** | **Taqman Assay ID** |
| --- | --- | --- | --- | --- |
| *Alox12*^a^ | CTTTGGTCCTGATGGCAAC | GACAATCAGGCCCAGGAGT | 105 | N/A |
| *Alox15*^a^ | GGGGATGGAGAAGCTACAGG | TCCGCTTCAAACAGAGTGC | 53 | N/A |
| *β-actin*^a^ | TGACAGGATGCAGAAGGAGA | CGCTCAGGAGGAGCAATG | 106 | N/A |
| *ALOX12*^b^ | - | - |  | Hs00167524_m1 |
| *ALOX15*^b^ | - | - |  | Hs00993765_g1 |
| *ALOX5*^b^ | - | - |  | Hs00167536_m1 |
| *HPRT1*^b^ | - | - |  | Hs02800695_m1 |

^a^Mouse genes, and ^b^Human genes.

N/A, Not Applicable


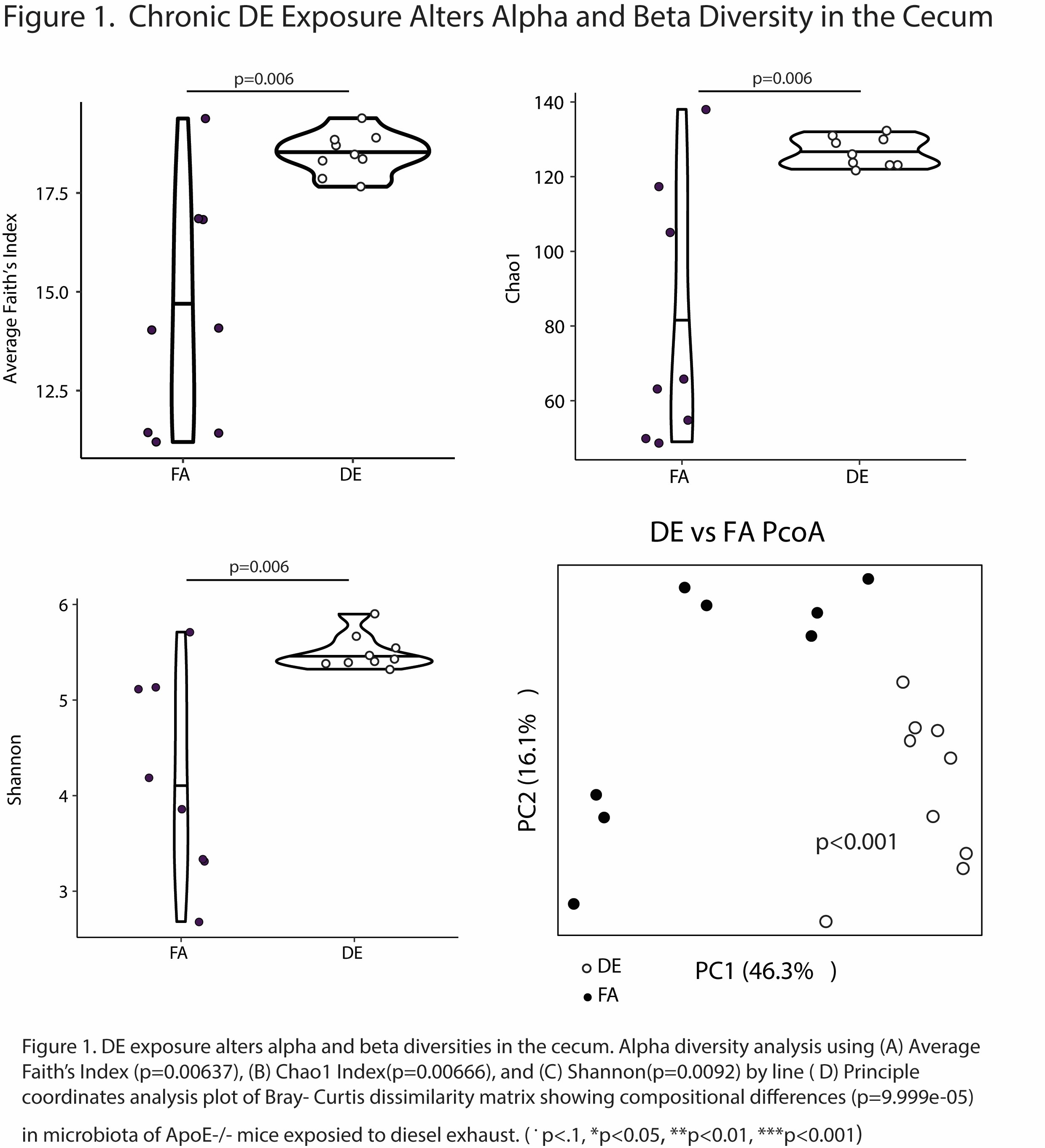

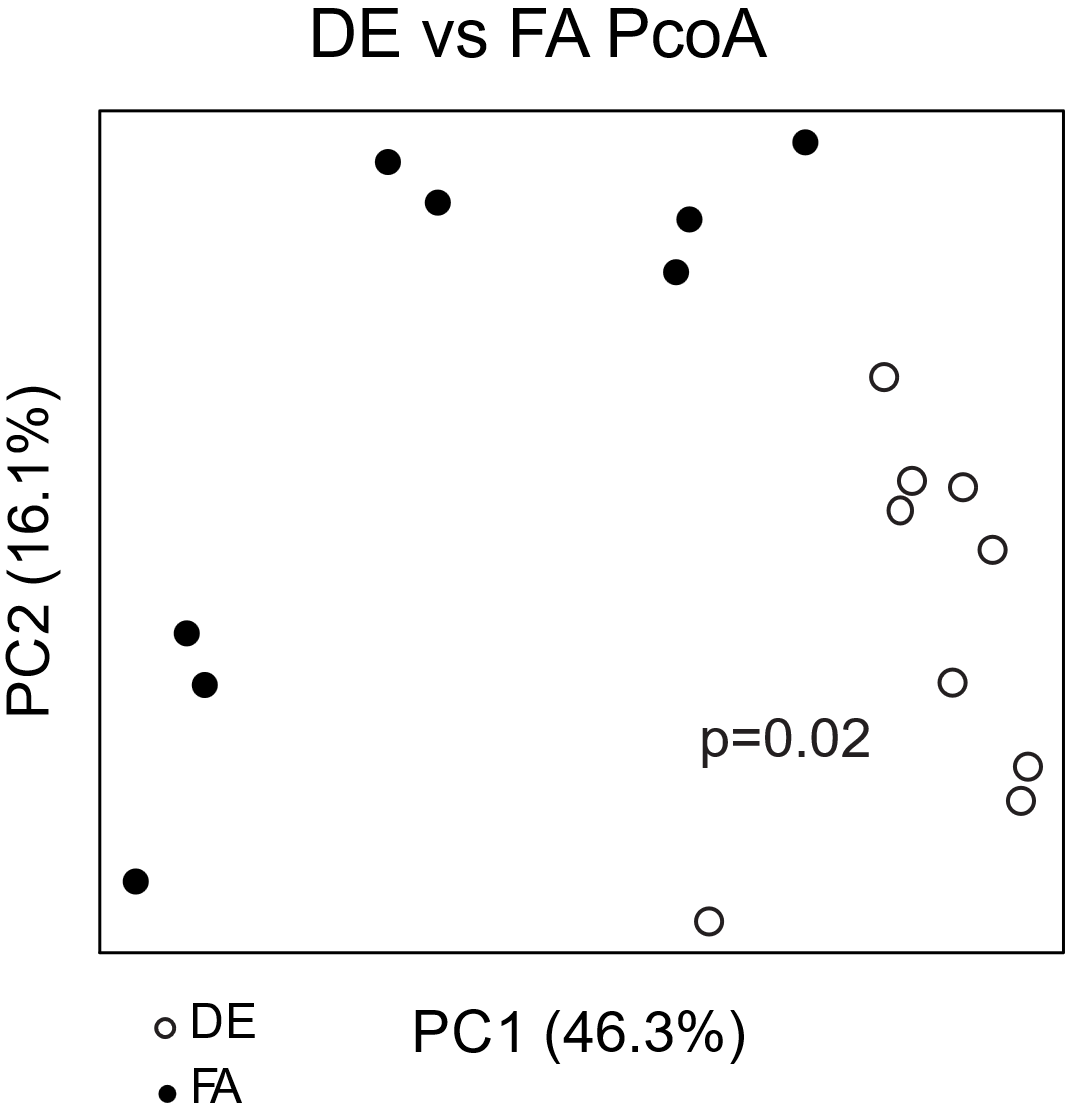


**B.**

**A.**


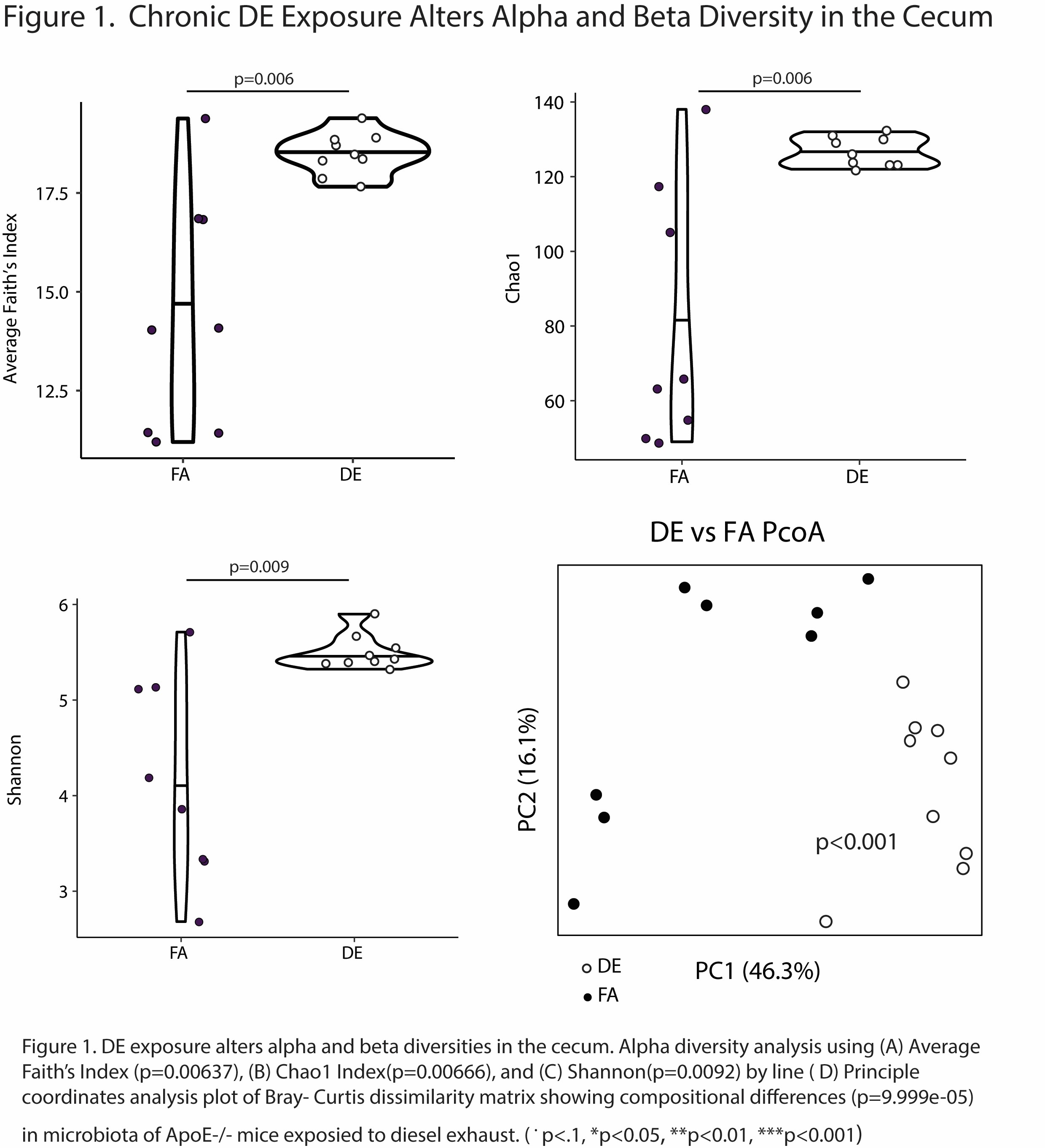


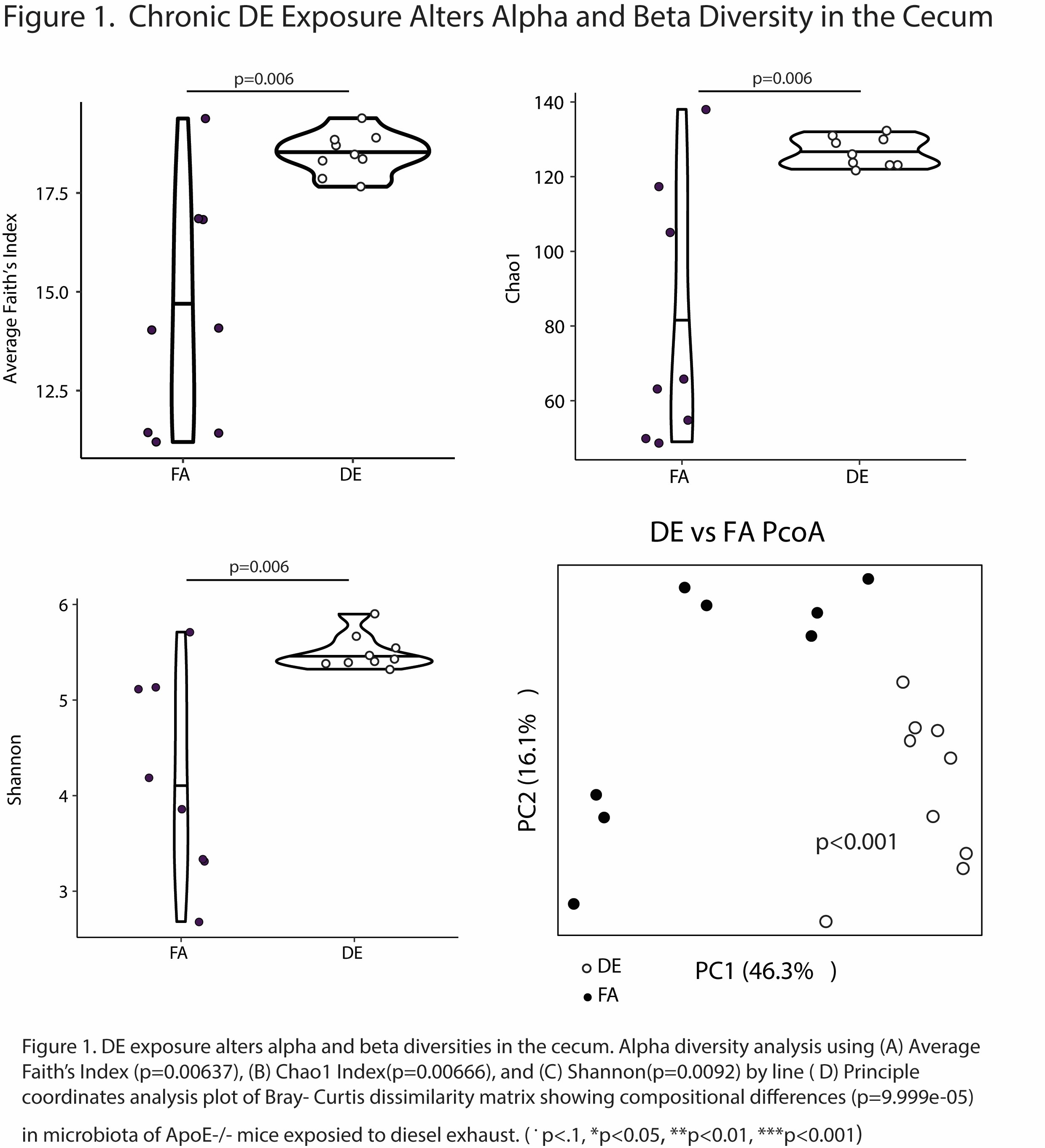


**D.**

**C.**

**Figure S1. DE exposure increased α- and β-diversity in the cecum before adjustment for cage effects. (A)** β-diversity, by principal coordinates analysis (PCoA) of Bray-Curtis dissimilarity. α-diversity using **(B)** Average Faith’s index, **(C)** Chao1 and **(D)** Shannon indices. Significance was assessed by t-test using Satterthwaite’s method and permutational multivariate analysis of variance (PERMANOVA), as described in the methods. n=8-9/group.


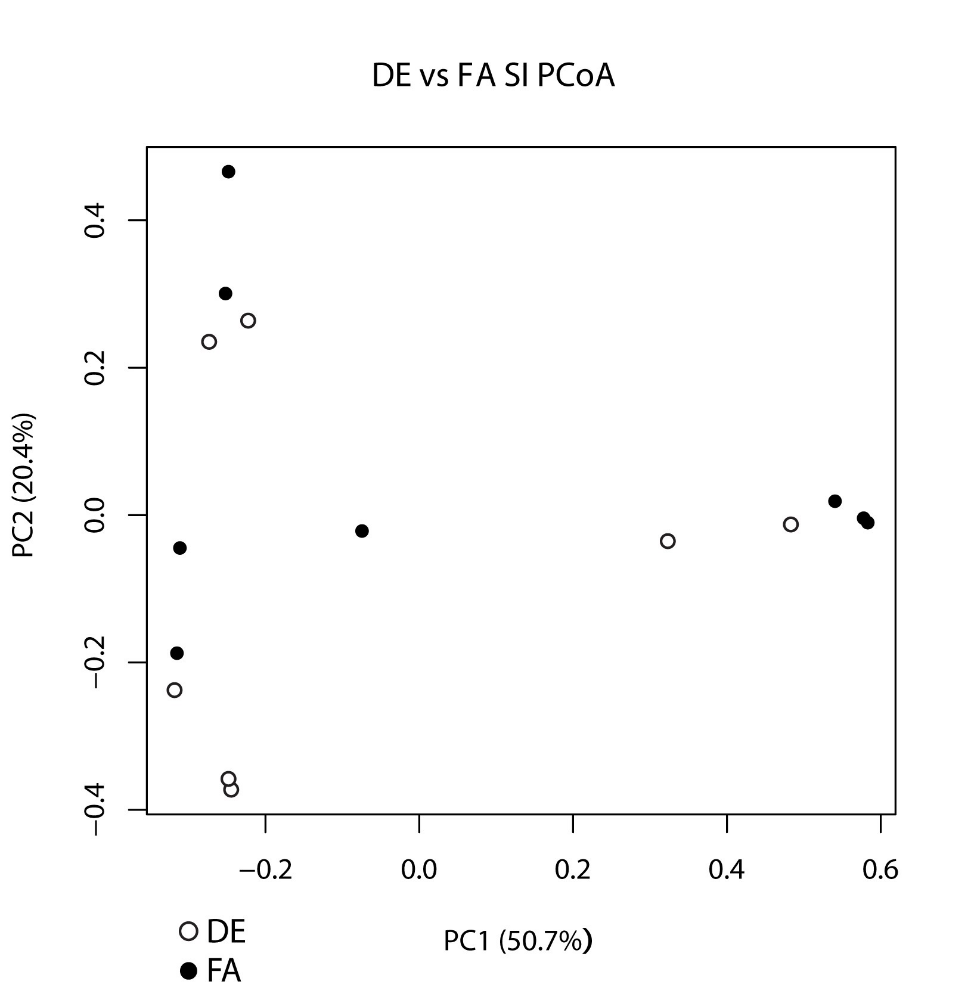


**Figure S2. β-diversity in the small intestine.** β-diversity was estimated using principal coordinates analysis (PCoA) of Bray-Curtis dissimilarity matrix showing compositional differences that did not reach statistical significance as assessed by PERMANOVA (p=0.08) in the intestinal microbiota of ApoE^-/-^ mice exposed to inhaled DE *vs.* FA. Each dot represents one sample with color representing the exposure group. n=7-8/group.


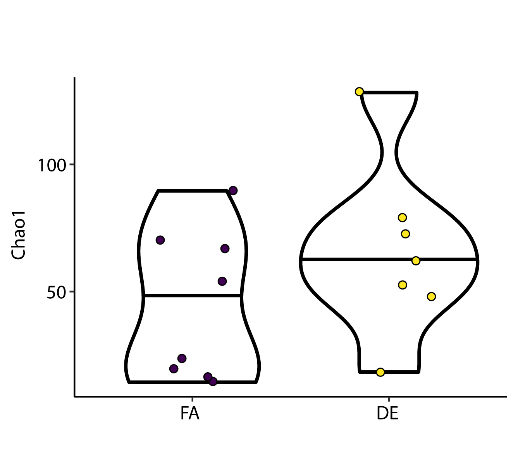


**C.**

**A.**

**B.**


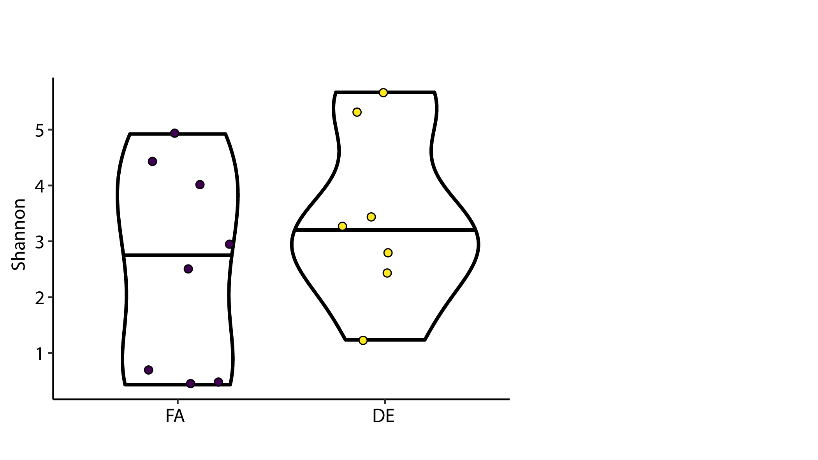

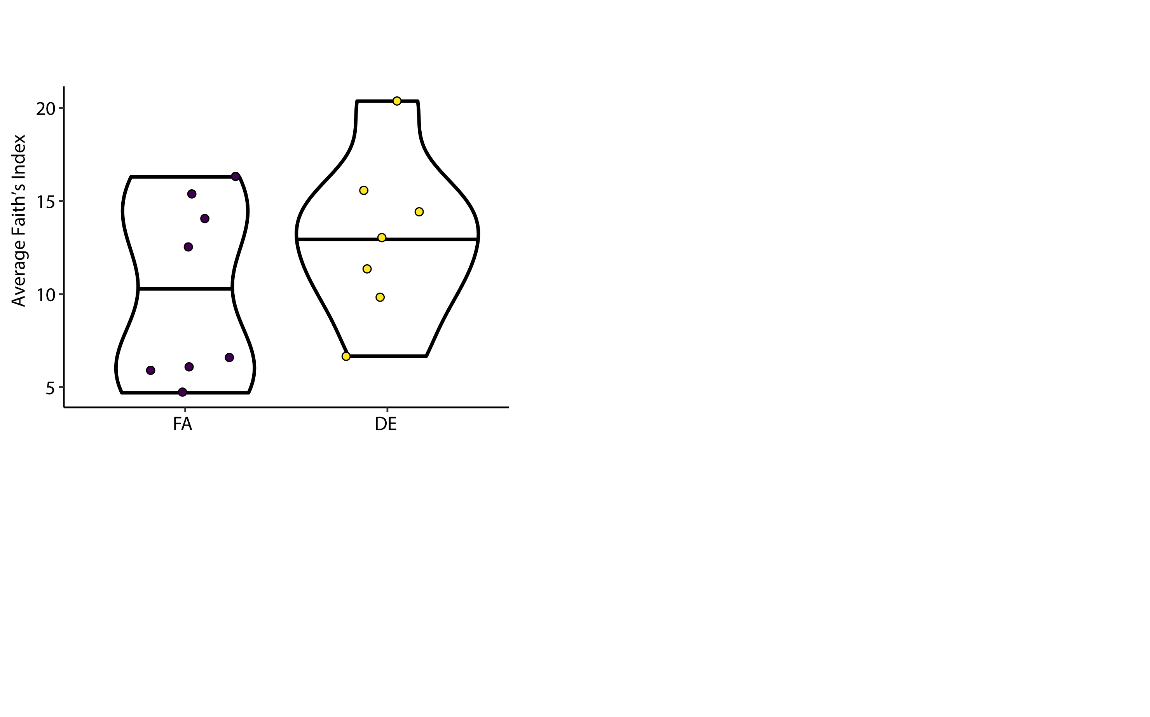


**Figure S3. α-diversity in the small intestine.** α-diversity analysis using **(A)** Average Faith’s index (p=0.45), **(B)** Chao1 (p=0.43) and **(C)** Shannon (p=0.49) indices, of the small intestinal microbiota in ApoE^-/-^ mice exposed to inhaled DE *vs.* FA. Each dot represents one sample with color representing the exposure group. n=7-8/group.


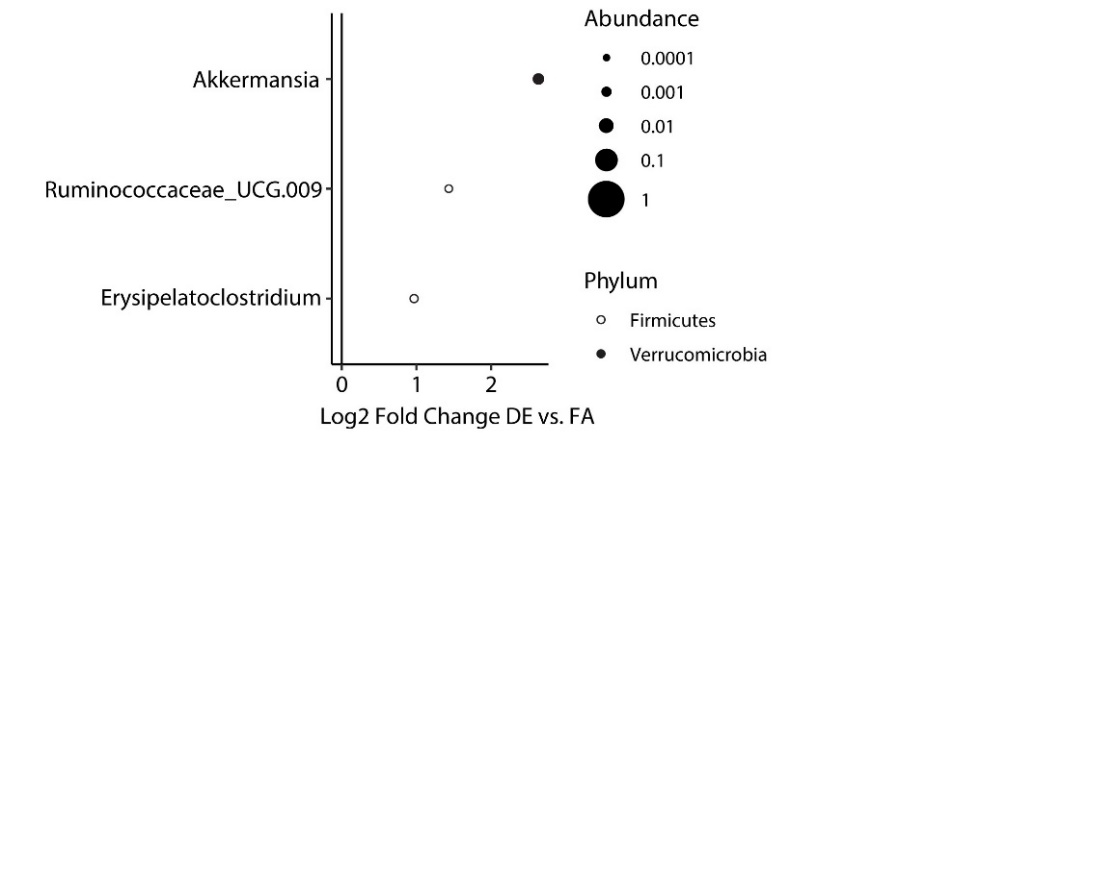


**B.**

**A.**

**C.**

**Figure S4. DE exposure altered the gut microbiota composition and abundance in the small intestine.** 16S rRNA sequencing of the DNA isolated from the contents of the small intestine revealed **(A)** significant differences in log2 fold change values of microbial taxa, and **(B-C)** differences in the %relative abundance of small intestinal bacteria at the phylum level in mice exposed to inhaled DE *vs.* FA. Phyla with >1% mean abundance were included. Dot size is proportional to the relative abundance of bacteria and color represents the phylum. The magnitude of effect is shown as the log2 of the fold change (Log2FoldChange) between the DE and FA exposed groups. n=7-8/group.

**A.**

**B.**

**Figure S5. Plasma Lipids.** Plasma levels of **(A)** total cholesterol and **(B)** triglycerides, among the DE and FA exposed groups. Data was taken from our previous study (Yin et al., 2019). Each bar denotes mean ± SEM (n=8/group). **p<0.01, DE *vs.* FA using Mann-Whitney U test (panel **(A)**) and *p<0.05, DE *vs.* FA using unpaired Student’s t-test (panel **(B)**).

**B.**

**A.**

**Figure S6. Hepatic triglycerides and oxidized fatty acids.** Hepatic levels of **(A)** triglycerides and **(B)** total HODEs (9-HODE + 13-HODE), among the DE and FA exposed groups. Data was taken from our previous study (Yin et al., 2019). Each bar denotes mean ± SEM (n=8/group for panel **(A)** and n=5-6/group for panel **(B)**). *p<0.05, DE *vs.* FA using Mann-Whitney U test (panel **(A)**) and ***p<0.001, DE *vs.* FA using unpaired Student’s t-test (panel **(B)**). HODEs, hydroxyoctadecadienoic acids.

**A.**

**C.**

**B.**

**D.**

**F.**

**E.**

**Figure S7. DE-induced alteration in cecal microbiome was significantly associated with hepatic 9- and 13-HODEs.** Inhaled DE *vs.* FA exposure led to significantly increased **(A)** Liver 9-HODE that were either **(B)** significantly positively associated or **(C)** inversely correlated with relative abundance of cecal bacteria at the genus level. **(D)** Liver 13-HODE were also elevated that were either **(E)** significantly positively associated or **(F)** inversely correlated with relative abundance of cecal bacteria at the genus level in ApoE^-/-^ mice exposed to inhaled DE *vs.* FA. **p<0.01, ***p<0.001 DE *vs.* FA using unpaired Student’s t-test. Spearman-correlation coefficients and *p* values are shown. n=5-6/group. HODE, Hydroxyoctadecadienoic acid.

**B.**

**A.**

**Figure S8. DE-induced reduction in fecal acetate levels significantly associated with changes in cecal microbiota composition. (A)** Spearman’s rank correlation indicating a positive association or **(B)** negative association between fecal acetate levels and cecal microbiome abundance at the genus level in ApoE^-/-^ mice exposed to inhaled DE *vs.* FA. Spearman-correlation coefficients and *p* values are indicated above.

**A.**

**B.**

**Figure S9. Fecal acetate levels and their association with liver total HODEs.** Inhaled DE *vs.* FA exposure led to significantly reduced levels of fecal acetate that were significantly negatively associated with **(A)** liver 9-HODE and **(B)** liver 13-HODE in ApoE^-/-^ mice. Pearson’s-correlation coefficients and *p* values are as indicated. n=5-6/group. HODE, Hydroxyoctadecadienoic acid.

**Figure S10. Mouse weights.** Representation of mouse weights after 16 weeks of exposure to FA and DE. Each bar denotes mean ± SEM (n=9-10/group). Data were analyzed using two-tailed Student’s t-test.

**References**

Anderson, M. J. (2014). Permutational multivariate analysis of variance (PERMANOVA). *Wiley statsref: statistics reference online*, 1-15.

Brooks, M. E., et al. (2017). glmmTMB balances speed and flexibility among packages for zero-inflated generalized linear mixed modeling. *The R journal, 9*(2), 378-400.

Callahan, B. J., et al. (2016). DADA2: High-resolution sample inference from Illumina amplicon data. *Nature methods, 13*(7), 581-583.

Gould, T., et al. (2008). A controlled inhalation diesel exhaust exposure facility with dynamic feedback control of PM concentration. *Inhal Toxicol, 20*(1), 49-52. doi:10.1080/08958370701758478

Livak, K. J., & Schmittgen, T. D. (2001). Analysis of relative gene expression data using real-time quantitative PCR and the 2(-Delta Delta C(T)) Method. *Methods, 25*(4), 402-408. doi:10.1006/meth.2001.1262

Rogers, G. W., et al. (2011). High throughput microplate respiratory measurements using minimal quantities of isolated mitochondria. *PLoS One, 6*(7), e21746. doi:10.1371/journal.pone.0021746

Satterthwaite, F. E. (1946). An Approximate Distribution of Estimates of Variance Components. *Biometrics Bulletin, 2*(6), 110-114. doi:10.2307/3002019

Yin, F., et al. (2019). Diesel Exhaust Induces Mitochondrial Dysfunction, Hyperlipidemia, and Liver Steatosis. *Arterioscler Thromb Vasc Biol, 39*(9), 1776-1786. doi:10.1161/ATVBAHA.119.312736

Yin, F., et al. (2013). Diesel exhaust induces systemic lipid peroxidation and development of dysfunctional pro-oxidant and pro-inflammatory high-density lipoprotein. *Arterioscler Thromb Vasc Biol, 33*(6), 1153-1161. doi:10.1161/ATVBAHA.112.300552
